# Epigenetic reader ZMYND11 noncanonical function restricts HNRNPA1-mediated stress granule formation and oncogenic activity

**DOI:** 10.1038/s41392-024-01961-7

**Published:** 2024-09-28

**Authors:** Cheng Lian, Chunyi Zhang, Pan Tian, Qilong Tan, Yu Wei, Zixian Wang, Qin Zhang, Qixiang Zhang, Mengjie Zhong, Li-Quan Zhou, Xisong Ke, Huabing Zhang, Yao Zhu, Zhenfei Li, Jingdong Cheng, Gong-Hong Wei

**Affiliations:** 1 Fudan University Shanghai Cancer Center & MOE Key Laboratory of Metabolism and Molecular Medicine and Department of Biochemistry and Molecular Biology of School of Basic Medical Sciences, and Institutes of Biomedical Sciences, Shanghai Medical College, Fudan University, Shanghai, China; 2 State Key Laboratory of Cell Biology, CAS Center for Excellence in Molecular Cell Science, Shanghai Institute of Biochemistry and Cell Biology, Chinese Academy of Sciences, University of Chinese Academy of Sciences, Shanghai, China; 3 Department of Urology, Fudan University Shanghai Cancer Center, Shanghai, China; 4 Disease Networks Research Unit, Biocenter Oulu and Faculty of Biochemistry and Molecular Medicine, University of Oulu, Oulu, Finland; 5 Institute of Reproductive Health, Tongji Medical College, Huazhong University of Science and Technology, Wuhan, Hubei China; 6 Shanghai Frontiers Science Center of TCM Chemical Biology, Institute of Interdisciplinary Integrative Medicine Research, Shanghai University of Traditional Chinese Medicine, Shanghai, China; 7 Department of Biochemistry and Molecular Biology, Metabolic Disease Research Center, School of Basic Medicine, Anhui Medical University, Hefei, China; 8 Minhang Hospital & Institutes of Biomedical Sciences, Shanghai Key Laboratory of Medical Epigenetics, International Co-laboratory of Medical Epigenetics and Metabolism, Fudan University, Shanghai, China; 9 State Key Laboratory of Common Mechanism Research for Major Diseases, Suzhou Institute of Systems Medicine, Chinese Academy of Medical Sciences & Peking Union Medical College, Suzhou, Jiangsu China

**Keywords:** Cancer genetics, Oncogenes

## Abstract

Epigenetic readers frequently affect gene regulation, correlate with disease prognosis, and hold significant potential as therapeutic targets for cancer. Zinc finger MYND-type containing 11 (ZMYND11) is notably recognized for reading the epigenetic marker H3.3K36me3; however, its broader functions and mechanisms of action in cancer remain underexplored. Here, we report that ZMYND11 downregulation is prevalent across various cancers and profoundly correlates with poorer outcomes in prostate cancer patients. Depletion of ZMYND11 promotes tumor cell growth, migration, and invasion in vitro, as well as tumor formation and metastasis in vivo. Mechanistically, we discover that ZMYND11 exhibits tumor suppressive roles by recognizing arginine-194-methylated HNRNPA1 dependent on its MYND domain, thereby retaining HNRNPA1 in the nucleus and preventing the formation of stress granules in the cytoplasm. Furthermore, ZMYND11 counteracts the HNRNPA1-driven increase in the PKM2/PKM1 ratio, thus mitigating the aggressive tumor phenotype promoted by PKM2. Remarkably, ZMYND11 recognition of HNRNPA1 can be disrupted by pharmaceutical inhibition of the arginine methyltransferase PRMT5. Tumors with low ZMYND11 expression show sensitivity to PRMT5 inhibitors. Taken together, our findings uncover a previously unexplored noncanonical role of ZMYND11 as a nonhistone methylation reader and underscore the critical importance of arginine methylation in the ZMYND11-HNRNPA1 interaction for restraining tumor progression, thereby proposing novel therapeutic targets and potential biomarkers for cancer treatment.

## Introduction

Cancer is a complex disease characterized by the intricate interactions between oncogenes and tumor suppressors, which are pivotal in tumor initiation, progression, and therapeutic response.^1^ Central to these processes are epigenetic modifications that regulate gene expression and maintain cellular identity. Aberrant epigenetic modifications, leading to dysregulation of the epigenome, have been strongly implicated in the development and progression of cancer. This dysregulation is orchestrated through a complex interplay among epigenetic “readers,” “writers,” and “erasers,” which collectively modulate chromatin landscape and gene expression.^2^ ZMYND11 (also known as BS69) is a notable epigenetic reader that selectively recognizes the trimethylation of lysine 36 on histone H3.3 (H3.3K36me3) through its PHD-bromo-PWWP (PBP) domain. Mutations and dysfunctions of ZMYND11 have been frequently implicated in various diseases, including cancer.^3–6^ H3.3K36me3 is associated with active transcription enriched within gene bodies,^7,8^ with the H3.3 histone variant peaking in active chromatin regions.^9^ Interestingly, evidence has emerged that ZMYND11 primarily suppresses gene expression by modulating RNA polymerase II (Pol II) during the elongation phase of transcription.^4–6^ Furthermore, the histone-reading domain PBP is located at the N-terminus of ZMYND11, whereas the MYND domain at its C-terminus is primarily linked with the transcriptional repression of downstream oncogenes and interactions with specific transcription factors and splicing-related proteins.^5,10–12^ These findings highlight the multifaceted roles of ZMYND11 in interpreting histone modifications and regulating transcription through various cooperative proteins. This functional diversity raises the possibility that ZMYND11 may possess non-canonical functions, including unconventional activities and antagonism toward key oncoproteins, which could be crucial in inhibiting cancer progression.

Cellular stress responses are increasingly recognized as critical contributors to cancer development and progression.^13^ One key aspect of these responses is the reprogramming of mRNA translation, a mechanism that enables cells to adapt and survive under adverse conditions.^14^ Emerging evidence highlights the role of stress granules (SGs) in regulating gene expression and protein translation, processes that are essential for cancer cell survival and proliferation.^14^ Stress granules are dynamic, membraneless organelles that transiently assemble in the cytoplasm of eukaryotic cells in response to various environmental stresses, such as heat shock, oxidative stress, and nutrient deprivation. Their formation is primarily driven by the accumulation of untranslated mRNAs and associated proteins during cellular stress.^15^ Aberrant stress granules have been implicated in the pathogenesis of several neurodegenerative diseases, including frontotemporal dementia and amyotrophic lateral sclerosis, due to their potential role in toxic protein aggregation and nucleation.^16^ In the context of cancer, stress granules confer survival advantages to malignant cells, contributing to chemotherapy resistance and acting as key players in various human disorders, including cancers.^17–19^ Recent studies suggest that stress granules function as dynamic systems capable of integrating oncogenic signals and tumor-related stress stimuli, thereby enhancing cancer cell fitness.^20^ Despite the significant advances in our understanding of stress granules and their role in cellular stress responses, the precise molecular interactions and mechanisms governing their assembly and disassembly remain largely elusive.

The formation of stress granules is a highly dynamic process, primarily driven by specific determinants located within the arginine–glycine–glycine repeat (RGG) domain of certain RNA-binding proteins (RBPs).^21^ Both asymmetrically and symmetrically dimethylated RGG domains are known to induce phase separation, leading to the formation of stress granules.^22–25^ The RGG domain-containing protein, heterogeneous nuclear ribonucleoprotein A1 (HNRNPA1), has been particularly noted for its ability to form stress granules under cellular stress conditions.^18^ These non-membrane-bound compartments assemble temporarily in response to stress and have been associated with poor disease prognosis.^26–28^ HNRNPA1 plays a fundamental role in gene expression and functions as a key RBP. It harbors two RNA recognition motifs (RRM) and a glycine-rich domain, which includes several Arg–Gly–Gly (RGG) tripeptide repeats. These structural elements enable HNRNPA1 to mediate cellular compartmentalization, protein-protein interactions, and RNA-binding.^29–31^ Despite the established importance of HNRNPA1 in these processes, the molecular mechanisms governing its oncogenic functions in cancer remain largely elusive. Furthermore, no therapeutic strategies currently exist that directly targets HNRNPA1. Given the association of stress granules with chemotherapy resistance, targeting their assembly could offer a promising therapeutic strategy to overcome both primary and acquired resistance, thereby enhancing treatment efficacy.^27,28,32,33^ However, the precise mechanisms underlying stress granule formation are not fully understood, and the role of HNRNPA1 in driving oncogenic processes in cancer requires further elucidation.

The methylation of arginine residues within the RGG domain is catalyzed by the family of arginine methyltransferases (PRMTs).^34,35^ Inhibitors of PRMTs are currently being explored as a promising therapeutic strategy for cancer, with various mechanisms proposed to explain the efficacy of PRMT inhibition.^36–40^ However, it remains unclear whether arginine methylations can be specifically recognized by certain protein readers and whether these interactions hold therapeutic potential. In the present study, we report that ZMYND11 plays a critical role in suppressing tumor cell proliferation, metabolism, and cancer progression through a noncanonical function. This function involves its interaction with PRMT5-mediated arginine methylation of HNRNPA1, thereby counteracting the oncogenic potential of HNRNPA1. Furthermore, we demonstrate that tumors with low ZMYND11 expression exhibit enhanced sensitive to inhibitors of the type II arginine methyltransferase PRMT5, highlighting a potential therapeutic avenue for targeting cancers with ZMYND11 dysregulation.

## Result

### ZMYND11 is profoundly downregulated in cancers and this downregulation correlates with adverse events and poor outcomes in prostate cancer patients

**Fig. 1:**
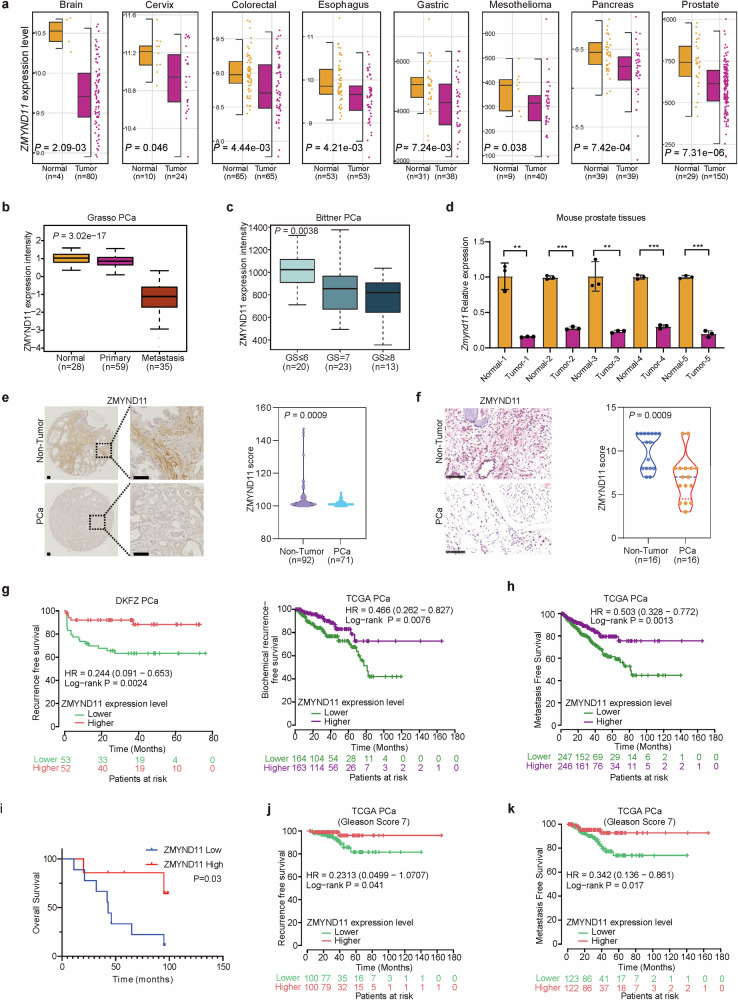
ZMYND11 is profoundly downregulated in cancers and its downregulation correlates with adverse events and poor prostate cancer patient outcomes. **a** Downregulation of *ZMYND11* gene expression in several types of cancers compared to healthy tissues (yellow). *P* values were assessed by the Wilcoxon test while effect sizes were estimated using Cohen’s distance: *d*_brain_ = 1.96, *d*_cervix_ = 0.86, *d*_colon_ = 0.56, *d*_esophagus_ = 0.64, *d*_gastric_ = 0.7, *d*_mesothelioma_ = 0.64, *d*_pancreas_ = 0.77, *d*_prostate_ = 0.99. **b** ZMYND11 mRNA expression was significantly downregulated in human metastatic prostate tumors. **c** Association of ZMYND11 downregulation with high Gleason grade cancer. *P* values examined by the Kruskal-Wallis test in (**b**, **c**). **d** Expression of *Zmynd11* in five paired prostate cancer specimens and adjacent normal tissues of murine prostate. **e** Protein expression levels of ZMYND11 in paraffinized prostate tumor tissue microarrays (TMA) of Tongji prostate cancer cohort determined by immunostaining. Original magnification, 100×; insets, 400×; Scale bar, 100 μm. **f** ZMYND11 protein expression were determined in Fudan cohort of prostate cancer using immunohistochemistry. The IHC scores calculated with staining areas (see Methods). Original magnification, 400×; Scale bar, 100 μm. **g** Kaplan-Meier analysis of biochemical recurrence-free survival in prostate tumors with high or low levels of ZMYND11 in two independent cohorts of prostate cancer. **h** Metastasis free survival analysis of 493 prostate cancer patient with tumors expressing high or low mRNA levels of ZMYND11. **i** Kaplan-Meier overall-survival analysis in a TMA cohort of patients with prostate cancer tumors having higher protein expression levels of ZMYND11 (top 50%; *n* = 35) or lower (bottom 50%; *n* = 35). Lower expression levels of ZMYND11 indicates predictive values for recurrence (**j**) and metastasis-free (**k**) survival in patient group with Gleason score 7 (intermediate-risk prostate cancer). In (**d**–**f**), statistical significance assessed using the two-tailed Student’s *t* test. **p < 0.01, ***p < 0.001. In (**g**–**k**), *p* values examined by the log-rank test

To gain comprehensive insight into the dysregulation of the epigenetic reader ZMYND11 in cancers, we initially examined the expression of ZMYND11 across various human tumor types and adjacent normal tissue samples. Our results revealed a clear and frequent downregulation of ZMYND11 in non-epithelial cancers (brain, mesothelioma) and in multiple epithelial cancer types (cervix, colorectal, esophagus, stomach, pancreas, prostate), with a particularly significant reduction observed in prostate cancer (Fig. 1a). This observation motivated us to further investigate the clinical relevance and consequences of ZMYND11 downregulation in prostate cancer, the most common and lethal urological cancer in men. We subsequently verified the downregulation of ZMYND11 using data from The Cancer Genome Atlas prostate Adenocarcinoma (TCGA PRAD) (Supplementary Fig. 1a). Importantly, ZMYND11 was consistently downregulated in metastatic samples from several independent human prostate cancer clinical datasets^41–44^ (Fig. 1b and Supplementary Fig. 1b), and its downregulation was notably correlated with high Gleason scores, advanced tumor stages, and elevated prostate-specific antigen (PSA) levels^45–47^ (Fig. 1c and Supplementary Fig. 1c). To further elucidate the importance of ZMYND11 in prostate cancer, we compared its expression at both mRNA and protein levels in prostate tissue lysates from mice with *Pbi–Cre*–mediated deletion of *Pten (Pten*^*−/−*^*)*, which develop indolent prostate cancer. As anticipated, ZMYND11 expression was significantly lower in murine prostate cancer tumors than in normal prostate glands (Fig. 1d and Supplementary Fig. 1d). Extending this observation, we performed immunohistochemistry (IHC) staining for ZMYND11 on two independent tumor tissue microarrays (TMA) using an antibody against ZMYND11. The results demonstrated a marked decrease in ZMYND11 protein levels in prostate cancer patient tumor samples compared to adjacent non-tumor tissues (Fig. 1e, f). Taken together, these findings suggest a potential role for ZMYND11 in restricting prostate cancer progression to advanced stage and metastasis.

To further explore the clinical significance of these findings, we conducted a prognosis correlation analysis across multiple independent cohorts of prostate cancer patients with long-term clinical outcomes. We discovered that patients with lower mRNA expression of ZMYND11 exhibited an increased risk of postoperative biochemical relapse, tumor progression to metastasis, and a shorter overall survival time^42,45,47^ (Fig. 1g, h and Supplementary Fig. 1e, f). Consistent with these findings, a Kaplan-Meier analysis of our TMA immunostaining results showed that prostate cancer patients with lower protein levels of ZMYND11 had a significant shorter overall survival time compared to patients with higher levels of ZMYND11 expression (Fig. 1i). Considering the consistent association between ZMYND11 expression status and prostate cancer prognosis, we investigated whether ZMYND11 levels could predict outcomes for low- and high-risk prostate cancer. We found that ZMYND11 mRNA levels could significantly predict biochemical recurrence and metastasis in patients with a Gleason score 7 (intermediate risk; Fig. 1j, k and Supplementary Fig. 1g), but not in patients groups with Gleason score ≤6 (low risk; Supplementary Fig. 1h) or ≥8 (high risk; Supplementary Fig. 1i). This indicates that ZMYND11 may serve as a potential prognostic marker to stratify intermediate-risk prostate cancer patients who may recur or progress to metastasis. Together, these data demonstrate that ZMYND11 downregulation frequently occurs during prostate cancer tumor progression to an advanced stage and correlates with an unfavorable prognosis in prostate cancer patients, suggesting that ZMYND11 could act as a tumor suppressor and that its reduced expression may contribute to prostate cancer tumorigenesis and metastasis.

### Dampened ZMYND11 expression promotes prostate cancer cell proliferation and metastasis in vitro and in vivo

**Fig. 2:**
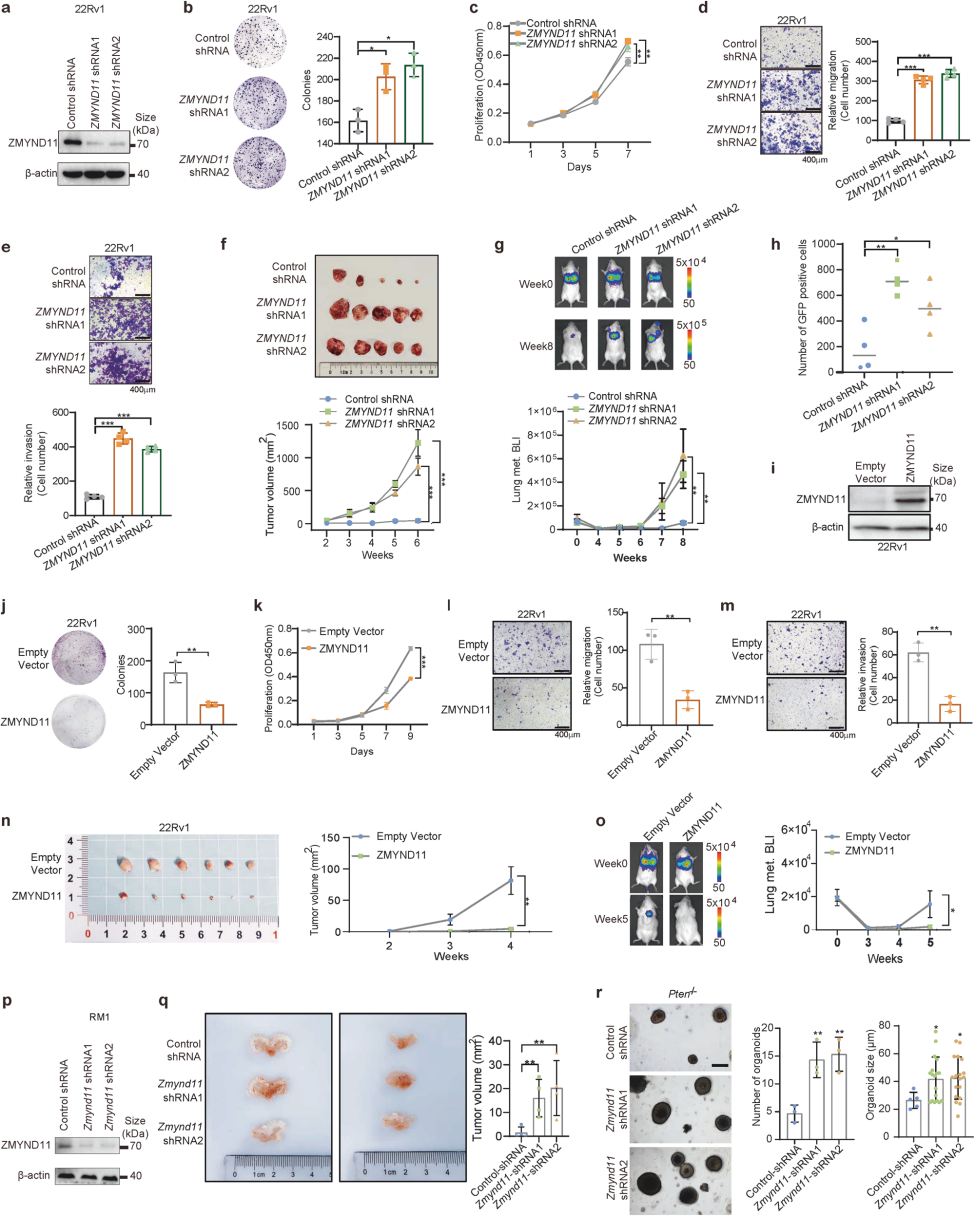
Dampened ZMYND11 expression promotes prostate cancer cell proliferation and metastasis in vitro and in vivo. **a** Efficiency of shRNA-mediated ZMYND11 knockdown was determined by immunoblotting. **b** Colony-forming units of 22Rv1 cells stably expressing control or shRNAs against ZMYND11. **c** Proliferation rates of 22Rv1 cells measured at indicated times (absorbance at 450 nm; mean ± SD of three independent experiments). Representative images and quantification of relative migration (**d**) and invasion (**e**) for the cells stably expressing the indicated shRNAs. **f** Representative images of the tumor xenografts harvested 6 weeks after subcutaneous injection of 1 × 10^7^ 22Rv1 cells with the indicated control or shRNA-mediated stable silencing of ZMYND11 into the nude mouse (left). Growth curves of 22Rv1 xenografts at the indicated time points. **g** NOD-SCID mice treated with tail vein injection of 22Rv1 cells having stable knockdown of ZMYND11 and stable transfection of the firefly luciferase. Representative images at the experimental endpoint and weekly quantitation of bioluminescent imaging (BLI) analyses of mouse lung metastasis. **h** Fluorescence activated cell sorting (FACS) analysis of the circulating GFP-positive 22Rv1 cell variants (CTCs) in the blood of SCID mice. The scatter plot shows the number of CTCs recovered from each mouse injected with control or shRNA-mediated ZMYND11 knockdown cells. **i** Ectopic expression efficiency of ZMYND11 was measured by Western blot analysis. **j** Colony-forming units of 22Rv1 cells ectopically expressing empty vector control or ZMYND11. **k** Proliferation rates of 22Rv1 cells measured at indicated times (absorbance at 450 nm; mean ± SD of three independent experiments). Representative images and quantification of relative migration (**l**) and invasion (**m**) for cells with ectopic expression of either the empty vector control or ZMYND11. **n** Representative images of tumor xenografts harvested four weeks after subcutaneous injection of 22Rv1 cells with either the control vector or ectopically expressing ZMYND11 into nude mouse (left). Showing on the right are the growth curves of the xenografts at the specified time points. **o** NOD-SCID mice treated with a tail vein injection of 22Rv1 cells ectopically expressing control vector or ZMYND11, along with stable transfection of firefly luciferase. Representative images at the experimental endpoint and weekly quantitation of bioluminescent imaging (BLI) analyses of mouse lung metastasis. **p** The efficiency of shRNA-mediated mouse Zmynd11 knockdown was determined by immunoblotting. **q** Representative images of tumor xenografts harvested 6 weeks after orthotopic implantation of 5 × 10^5^ RM1 cells with either control or shRNA-mediated stable silencing of ZMYND11 into the prostate of C57BL/6J mice (left). Tumor volume of xenografts at the 6-week endpoint is shown on the right. **r** Representative images of organoids derived from prostatic *Pten*^*−/−*^ mice with or without shZMYND11 treatment. Quantitative results from three experiments are shown on the right. Scale bar: 40 μm. In (**b**–**r**), data shown are mean ± SD Error bars, *p < 0.05, **p < 0.01, ***p < 0.001, two-tailed Student’s *t* test

We proceeded to examine the impact of ZMYND11 on prostate cancer tumor cellular phenotypes and invasiveness by conducting knockdown assays using lentivirus-mediated short hairpin RNA (shRNA) or synthetic small interfering RNA (siRNA) against ZMYND11 in several prostate cancer cell models (Fig. 2a and Supplementary Fig. 2a). The results consistently demonstrated that dampened ZMYND11 greatly stimulated colony formation, cell growth, migration, and invasion in the prostate cancer cell lines 22Rv1, DU145, and LNCaP (Fig. 2b-e and Supplementary Fig. 2b–e). We also assessed the effect of ZMYND11 knockdown on tumor growth in vivo in a xenograft mouse model. The findings indicated that xenograft prostate cancer tumors derived from 22Rv1 or DU145 cells with stable ZMYND11 silencing exhibited significantly enhanced growth compared to xenografts from control cells (Fig. 2f and Supplementary Fig. 2f). Additionally, we developed 22Rv1 or DU145 cell models stably expressing firefly luciferase for metastatic tumor growth assays and quantitated lung metastasis in a mouse model following tail vein injections using bioluminescent imaging (BLI). Our weekly BLI analysis revealed that ZMYND11 knockdown markedly promoted lung metastasis (Fig. 2g and Supplementary Fig. 2g). Further analysis using FACS to separate GFP-positive circulating cancer cells from the blood showed a significantly higher number of circulating 22Rv1 cells with ZMYND11 knockdown compared to controls (Fig. 2h). This suggests that cells with reduced ZMYND11 expression have an enhanced survival capacity in the bloodstream, thereby facilitating metastasis to distant organs.

Next, to better understand the impact of ZMYND11 within the tumor microenvironment, we utilized the well-characterized orthotopic prostate cancer xenograft mouse model.^48^ This model is widely used to study the molecular events of primary tumor development and the cross-talk between the tumor and organ microenvironment. We first established mouse *Zmynd11* knockdown in RM1 cell lines, which originated from the prostate cancer of C57BL/6J mice (Fig. 2p), and implanted these cells orthotopically into the prostate of C57BL/6J male mice. As shown in Fig. 2q, knockdown of mouse *Zmynd11* increased prostate cancer cell growth compared with the control group, suggesting that ZMYND11 suppresses tumor growth in the tumor microenvironment. Additionally, we conducted an organoid formation assay to investigate the autonomous function of ZMYND11. The results indicated that reduced ZMYND11 expression enhanced the ability of tumor cells to form organoids (Fig. 2r).

Collectively, these findings underscore the critical role of ZMYND11 downregulation in promoting prostate cancer cell growth and metastasis both in vitro and in vivo, consistent with the observed association between low ZMYND11 levels, aggressive prostate tumor phenotype, prostate cancer progression, and poor prognosis in clinical settings.

### Overexpression of ZMYND11 inhibits prostate cancer cell proliferation and metastasis in vitro and in vivo

To examine the impact of upregulating ZMYND11 on prostate cancer cellular phenotypes and invasiveness, we conducted experiments in vitro and in xenograft mouse models in vivo. 22Rv1 and DU145 cells were transfected with either a control vector or ZMYND11 (Fig. 2i and Supplementary Fig. 2h). Consistent with the knockdown assays, overexpression of ZMYND11 significantly suppressed colony formation, cell growth, migration, and invasion in the prostate cancer cell lines 22Rv1 and DU145 (Fig. 2j–m and Supplementary Fig. 2i–l). Furthermore, stable overexpression of ZMYND11 inhibiting tumor growth and lung metastasis (Fig. 2n, o and Supplementary Fig. 2m, n). Taken together, upregulation of ZMYND11 reduces prostate cancer cell growth and metastasis both in vitro and in vivo.

### ZMYND11 interacts with HNRNPA1 through its MYND domain and inhibits HNRNPA1-mediated formation of stress granule

**Fig. 3:**
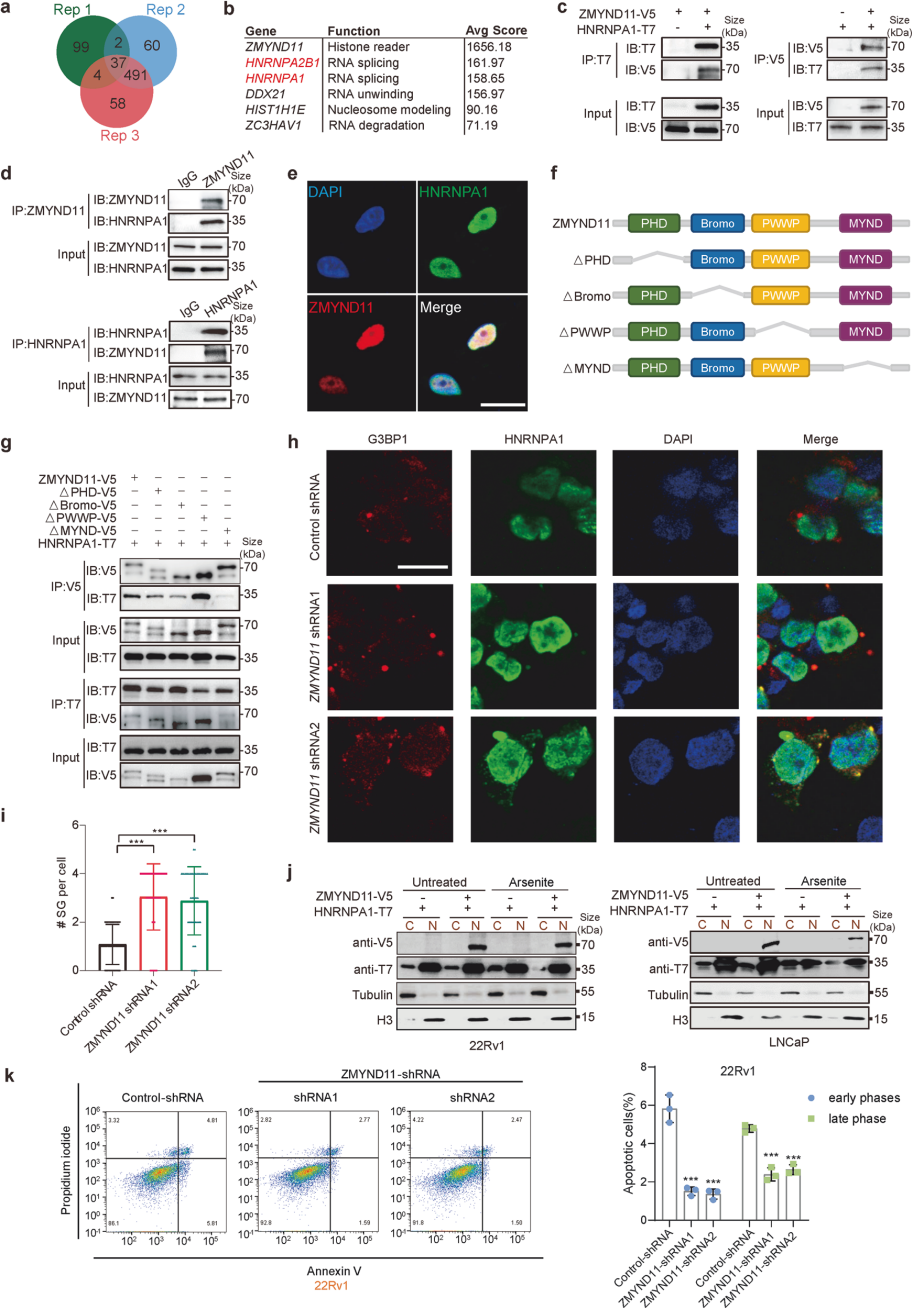
ZMYND11 physically interacts with HNRNPA1 in vitro and in vivo and inhibits HNRNPA1-mediated formation of stress granules. **a** Venn diagram showing the overlap between three replicates of the identified ZMYND11-interaction proteins in LNCaP cells using co-immunoprecipitation coupled to mass spectrometry (Co-IP/MS). **b** Table showing top-scoring ZMYND11-interaction proteins reproducibly identified by Co-IP/MS as indicated in (**a**). **c** Immunoprecipitation (IP)-Western blot for analyzing the interaction between ZMYND11 and HNRNPA1 in HEK293T cells cotransfected with the indicated V5- or T7-tagged plasmids. IB immunoblot. **d** Immunoprecipitation-Western blot analysis of an endogenous interaction of ZMYND11 with HNRNPA1 in 22Rv1 cells. IgG immunoglobulin, IB immunoblot. **e** Immunofluorescence staining and colocalization analysis of ZMYND11 and HNRNPA1 in 22Rv1 cells. Scale bar, 25 μm. **f** Schematic illustration of the full-length ZMYND11 and the indicated domain deletion mutants. **g** Immunoprecipitation-Western blot analysis of the interactions between wild type ZMYND11 or its mutants and HNRNPA1. Crude total cell lysates were extracted from HEK293T cells co-expressing T7-tagged HNRNPA1 and the indicated V5-tagged ZMYND11 variants. **h** 22Rv1 cells stably expressing control or shRNAs against ZMYND11, treated with sodium arsenite (SA, 0.5 mM) for 1 h followed by immunostaining analysis of stress granule (SG) formation. Scale bar, 25 μm. **i** The number of SGs per cell and ZMYND11 or HNRNPA1 colocalized SGs each cell were quantified. **p < 0.01, ***p < 0.001 evaluated by two-tailed Student’s *t* test. **j** Immunoblotting analysis of ZMYND11 or HNRNPA1 in the nuclear and cytoplasmic fractions of the tested prostate cancer cell lines 22Rv1 and LNCaP, respectively. **k** Apoptosis of 22Rv1 cells stably expressing control or shRNAs targeting ZMYND11. ***P < 0.001 assessed by two-tailed Student’s *t* test

To uncover the molecular mechanisms through which ZMYND11 regulates cancer cell growth and metastasis, we aimed to identify new proteins interacting with ZMYND11. Therefore, we conducted an immunoprecipitation experiment in prostate cancer LNCaP cells using an antibody against endogenous ZMYND11, followed by mass spectrometry (MS) analysis of the precipitated proteins. In three independent experiments, a total of 37 proteins were robustly identified as associated with ZMYND11 (Fig. 3a). Gene Ontology (GO) analysis revealed that the majority of the proteins associated with ZMYND11 are enriched in the functional category of RNA splicing (Supplementary Fig. 3a), aligning with previous findings that ZMYND11 regulates RNA alternative splicing (AS).^4,5^ Among the candidate ZMYND11 interaction proteins, HNRNPA2B1 and HNRNPA1 emerged as the top hits in three repeated protein complex identifications (Fig. 3b). Subsequent co-immunoprecipitation (Co-IP) assays confirmed that HNRNPA1, but not HNRNPA2B1, interacts with ZMYND11 in transfected cells (Fig. 3c and Supplementary Fig. 3b). This physiological interaction between ZMYND11 and HNRNPA1 was verified at endogenous levels in 22Rv1 cells (Fig. 3d) and through immunofluorescence assay, which showed colocalization of endogenous ZMYND11 with HNRNPA1 in the nucleus (Fig. 3e). Additionally, ZMYND11 was found to interact with HNRNPA1 in the lung cancer cell line A549 and the breast cancer cell line MDA-MB-231 (Supplementary Fig. 3c), indicating that the association between ZMYND11 and HNRNPA1 might be common across different cancer types.

We then determined which domain of the ZMYND11 protein facilitated its interaction with HNRNPA1. Considering that ZMYND11 comprises plant homeodomain (PHD), bromodomain (Bromo), Pro-Trp-Trp-Pro (PWWP), and myeloid, Nervy, and DEAF-1 (MYND) domains, we generated four domain-deletion mutant constructs tagged with a C-terminal V5 (Fig. 3f). Co-IP experiments revealed that the deletion of the MYND domain, unlike the PWWP, Bromo, or PHD domain of ZMYND11, significantly reduced the binding of full-length ZMYND11 to HNRNPA1. This indicates that the MYND domain of ZMYND11 is critical for its interaction with HNRNPA1 (Fig. 3g and Supplementary Fig. 3d), suggesting that ZMYND11 interacting with HNRNPA1 is mediated by the MYND domain.

Both in vitro and in vivo evidence as described above support a direct interaction between the tumor suppressor ZMYND11 and the oncogenic HNRNPA1, prompting us to investigate the potential functional consequences of this novel protein association. Notably, HNRNPA1 has been implicated in the assembly of stress granules into liquid-like droplets through a process known as liquid-liquid phase separation, with stress granule formation playing roles in preventing cancer cell apoptosis, tumor progression and chemotherapeutic resistance.^20,27,33,49,50^ We therefore explored the role of the ZMYND11-HNRNPA1 association in stress granule dynamics by treating cells with sodium arsenite (SA; 0.5 mM, 1 h). Immunofluorescence analysis showed an increase in cytoplasmic-localized HNRNPA1 in cells exhibiting a constitutively active form of stress granules that colocalized with the stress granule marker G3BP1,^51,52^ whereas ZMYND11 as a control, could not assemble stress granules in response to SA treatment (Supplementary Fig. 3e, f). Upon SA treatment, we observed the endogenous behavior of HNRNPA1. Remarkably, ZMYND11 knockdown resulted in increased stress granule formation that co-localized with HNRNPA1 (Fig. 3h, i). When ZMYND11 and HNRNPA1 were simultaneously overexpressed in cells, the stress-induced cytoplasmic accumulation of HNRNPA1 and its stress granule formation were significantly inhibited (Supplementary Fig. 3g, h). Consistent with this, nuclear and cytoplasmic separation experiments demonstrated that cytoplasmic HNRNPA1 levels decreased, likely due to ZMYND11 physical association with and subsequent sequestration of HNRNPA1 in the nucleus under stress conditions (Fig. 3j).

However, stress granules can also suppress the apoptosis of cancer cells.^33,53^ To investigate whether the tumor suppressive roles of ZMYND11 are due to stress granules-induced apoptosis, we analyzed the apoptosis rate of control and ZMYND11 knockdown groups after 1 h of treatment with SA, using Annexin V-488 and PI double-stained flow cytometry. Compared to the control, the proportion of early apoptotic cells in ZMYND11 knockdown 22Rv1 cells significantly decreased from 5.81% to 1.59% and 1.50% (*p* < 0.05), and in DU145 cells from 5.95% to 0.62% and 0.66% (*p* < 0.05). This suggests that ZMYND11 can induce early apoptosis in prostate cancer under stress conditions. Additionally, the proportion of late-apoptotic cells also decreased upon ZMYND11 knockdown, from 4.81% to 2.77% and 2.47% in 22Rv1 cells, and from 6.50% to 0.80% and 0.70% in DU145 cells, respectively, compared to the control group (Fig. 3k and Supplementary Fig. 3i). These results confirm that ZMYND11 exhibits tumor suppressive roles by preventing the formation of stress granules and increasing apoptosis of cancer cells under stress conditions.

Collectively, these findings suggest that ZMYND11 interacts with HNRNPA1 via the MYND domain and can limit the cytoplasmic translocation of HNRNPA1 to stress granules in stressed cells, thereby increasing the apoptosis of cancer cells.

### ZMYND11 counters oncogenic effects of HNRNPA1-induced aggressive cellular phenotypes in prostate cancer through its MYND domain

**Fig. 4:**
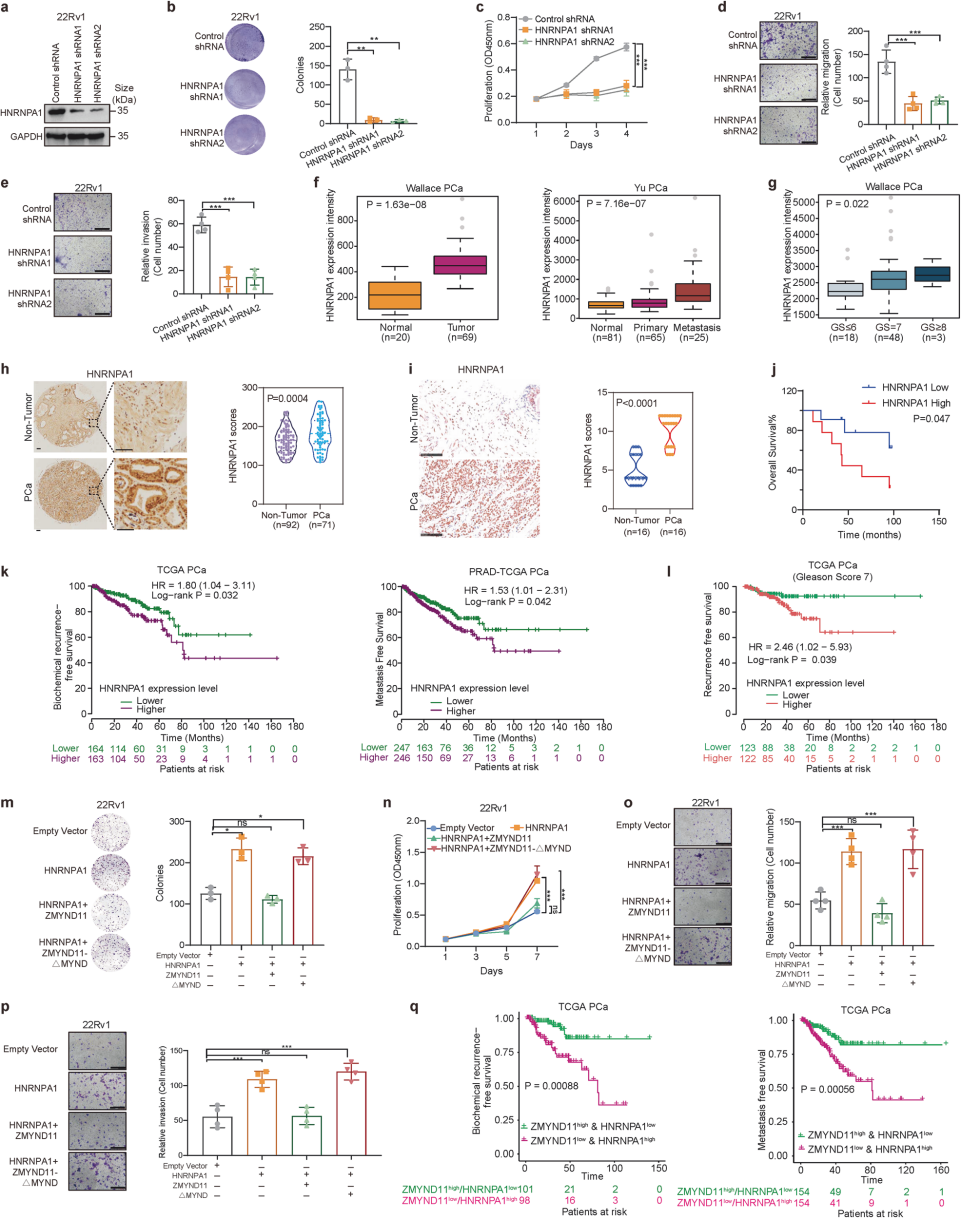
ZMYND11 antagonizes oncogenic function of HNRNPA1 in prostate cancer dependent on its MYND domain. **a** Knockdown efficiency of shRNAs against HNRNPA1 determined by western blot analysis. **b** Colony-forming units of 22Rv1 cells stably expressing control or shRNAs against HNRNPA1. **c** Proliferation capacity of 22Rv1 cells measured at the indicated times. Absorbance at 450 nm; mean ± SD of three independent experiments. Representative images and quantification of relative migration (**d**) and invasion (**e**) for the cells stably expressing the indicated shRNAs. Error bars, ±SD of triplicate experiments. **f** HNRNPA1 expression levels are significantly upregulated in human metastatic prostate tumors. *P* values determined by the Wilcoxon and Kruskal-Wallis tests, respectively. **g** HNRNPA1 upregulation correlates with higher Gleason grade cancer. *P* values assessed by the Kruskal-Wallis test. **h** Immunohistochemistry on the expression of HNRNPA1 and evaluation of HNRNPA1 staining intensity in a paraffinized prostate tissue microarray consisting of 163 samples. Original magnification, 100×; insets, 400×; Scale bar, 100 μm. **i** Immunostaining for HNRNPA1 was performed on another cohort of prostate specimens. Representative images and quantification of HNRNPA1 protein staining are shown. Original magnification, 400×; Scale bar, 100 μm. **j** Kaplan-Meier analysis of overall survival in the Tongji cohort of patients with prostate cancer. Patients were stratified into two groups according to the state of HNRNPA1 expression, higher (top 50%; *n* = 35) or lower (bottom 50%; *n* = 35). **k** Kaplan-Meier analysis showed that HNRNPA1 upregulation is significantly associated with elevated risk of biochemical recurrence or metastasis in prostate cancer subsets within the TCGA database. **l** High expression levels of HNRNPA1 indicates predictive values for recurrence-free survival in patient group with Gleason sum score 7 prostate cancer (intermediate-risk). Representative images and quantification of colony formation (**m**), cell proliferation (**n**), migration (**o**), and invasion (**p**) for 22Rv1 cells transfected with empty vector, HNRNPA1 alone, or together with the indicated full-length ZMYND11 or MYND-domain deletion mutant. Scale bar, 400 μm. **q** Kaplan-Meier analysis of biochemical recurrence or metastasis-free survival in the TCGA cohort of prostate cancer patients stratified into two groups with ZMYND11^low^/HNRNPA1^high^ and ZMYND11^high^/HNRNPA1^low^ expression, respectively. Error bars, ±SD (**b**–**e,** **m**–**p**). ns not significant, *p < 0.05, **p < 0.01, ***p < 0.001, two-tailed Student’s *t* tests

HNRNPA1 has been extensively studied across various human cancers, with relatively few reports in prostate cancer.^54–56^ We therefore investigated the impact of HNRNPA1 on tumor cellular phenotypes in prostate cancer and found that silencing HNRNPA1 through shRNA or siRNA led to inhibited cell growth, colony formation, migration, and invasion in prostate cancer cell lines 22Rv1, DU145, and LNCaP (Fig. 4a–e and Supplementary Fig. 4a–e). Accumulating evidence indicates that HNRNPA1 generally acts as an oncogene, with its upregulation associated with higher pathological stages and poorer prognosis in multiple cancer types.^56^ We assessed HNRNPA1 mRNA levels across several independent clinical datasets, revealing that HNRNPA1 expression was significantly upregulated during prostate cancer progression to advanced and metastatic stages in four cohorts^42,44,57,58^ (Fig. 4f and Supplementary Fig. 4f). Furthermore, HNRNPA1 expression was higher in patients with elevated Gleason scores and correlated with an increased risk of disease relapse and metastasis^44,47^ (Fig. 4g and Supplementary Fig. 4g). Protein levels of HNRNPA1 were also measured in prostate cancer tumors from mice with prostate-specific deletion of *Pten (Pbi–cre; Pten*^*loxP/loxP*^*)* compared to normal murine prostate glands, showing increased HNRNPA1 in tumor tissues (Supplementary Fig. 4h). Additionally, we evaluated HNRNPA1 protein levels in a collection of prostate cancer patient samples compared to surrounding non-tumor tissues using IHC, finding elevated HNRNPA1 expression in tumor specimens (Fig. 4h, i). Importantly, patients with higher HNRNPA1 levels had significantly shorter overall survival than those with lower HNRNPA1 expression (Fig. 4j). Analysis of several public datasets corroborated our findings, indicating that prostate cancer patients with higher HNRNPA1 levels experienced quicker disease relapse and metastasis^47,59^ (Fig. 4k and Supplementary Fig. 4l). Notably, HNRNPA1 expression was predictive of disease recurrence in prostate cancer patients with a Gleason score of 7 but not in those with Gleason scores ≤6 or ≥8 (Fig. 4l and Supplementary Fig. 4j), suggesting a prognostic potential of HNRNPA1 in patient stratification.

We subsequently explored the impact of ZMYND11 on HNRNPA1 function in prostate oncogenesis by conducting forced co-expression of full-length ZMYND11 or a MYND-domain-deleted variant with HNRNPA1 in prostate cancer cells. The findings revealed that full-length ZMYND11, but not the MYND-domain-deleted variant, counteracted the enhancement of cell growth, colony formation, migration, and invasion induced by HNRNPA1 overexpression (Fig. 4m–p and Supplementary Fig. 4k–n), suggesting that ZMYND11 mitigates the oncogenic function of HNRNPA1 through a physical interaction mediated by its MYND domain. Given the correlation between ZMYND11 downregulation or HNRNPA1 upregulation and adverse outcomes in prostate cancer patients, we then examined whether these observations have relevant clinical implications. As shown in Fig. 4q, patients with low ZMYND11/high HNRNPA1 expression exhibited significantly shorter biochemical recurrence- or metastasis-free survival compared to those with high ZMYND11/low HNRNPA1 expression, indicating a synergistic prognostic effect of both genes on prostate cancer severity. Altogether, our data elucidate the oncogenic and clinical impacts of HNRNPA1 on prostate cancer progression and demonstrate that ZMYND11 attenuates the oncogenic capabilities of HNRNPA1 through binding mediated by the MYND domain of ZMYND11.

### ZMYND11 modulates HNRNPA1-mediated alternative splicing of PKM and mitigates the aggressive phenotype of prostate cancer cells induced by PKM2

**Fig. 5:**
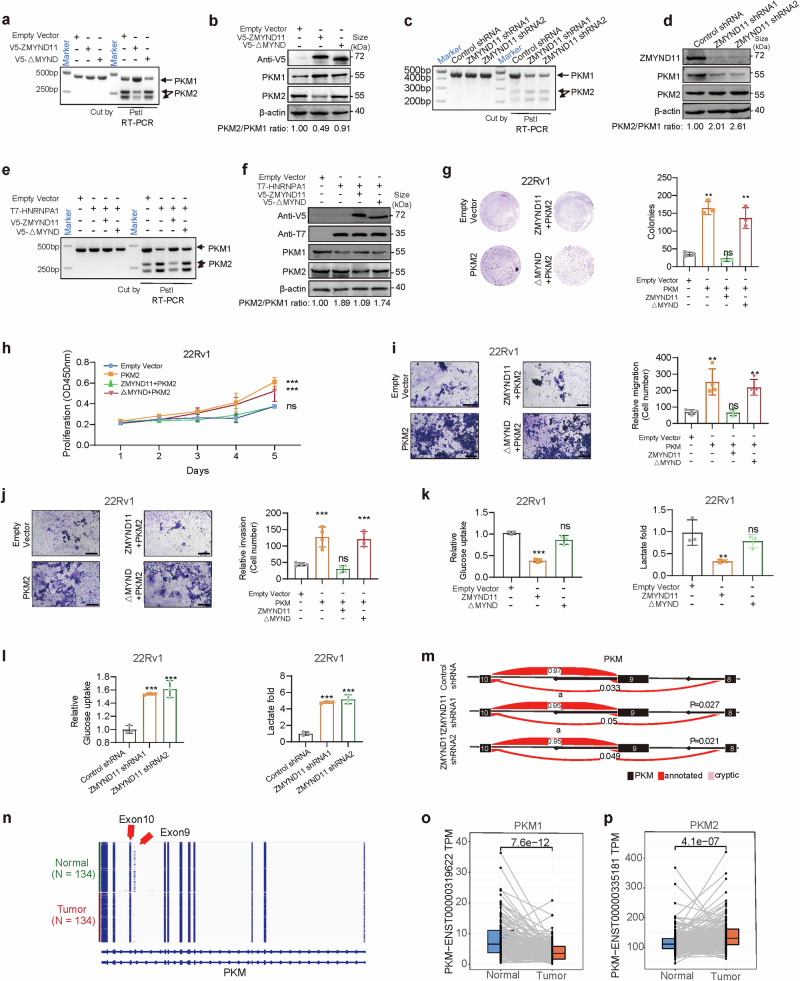
ZMYND11 regulates HNRNPA1-mediated alternative splicing of PKM and counteracts PKM2-induced aggressive cellular phenotype in prostate cancer. RNA (**a**, **c**) or protein (**b**, **d**) was extracted from 22Rv1 cells either transfected with full-length ZMYND11 or the indicated mutant (**a**, **b**) or stably expressing the ZMYND11-targeting shRNAs (**c**, **d**) followed by the examination of PKM isoforms both at the mRNA (**a**, **c**) and protein levels (**b**, **d**). 22Rv1 cells transfected with HNRNPA1 alone or together with either full-length ZMYND11 or the MYND-domain-deletion mutant were assayed for PKM splicing at the mRNA (**e**) and protein levels (**f**). Representative images and quantification of colony forming (**g**), cell proliferation (**h**), migration (**i**), and invasion (**j**) in 22Rv1 cells co-transfected with PKM2 alone or together with either the full-length ZMYND11 or the MYND-domain-deletion mutant. Mean ± SD of triplicate experiments. *p < 0.05, **p < 0.01, ***p < 0.001, Student’s *t* test. Scale bar, 400 μm. Relative glucose uptake and lactate production in 22Rv1 cells overexpressing ZMYND11 or its MYND-domain-deletion mutant (**k**) and stably expressing ZMYND11 shRNA (**l**) compared to control cells. Error bars, ±SD (**k**–**l**), *n* = 3 biological replicates. *p < 0.05, **p < 0.01, ***p < 0.001, Student’s *t* tests. **m** Visualization of PKM splicing using LeafViz examined by RNA sequencing in 22Rv1 cells stably expressing either ZMYND11-targeting or control shRNAs. Statistic assessment via two-tailed Student’s *t* test. **n** Visualization of PKM isoform expression in 134 patients of prostate tumors and matched normal tissues in CPGEA cohort by RNA-seq profiling [PMID: 32238934]. Differential expression analysis of PKM1 (**o**) or PKM2 (**p**) in this patient cohort of 134 tumor-normal paired prostate specimens. The data were examined by the Wilcoxcon test compares two paired groups

HNRNPA1 has been shown to facilitate the excision of exon 9 from the PKM pre-mRNA, thereby selectively inducing the formation of the oncogenic PKM2 isoform, a key determinant of the Warburg effect that promotes aerobic glycolysis and cancer cell survival^29–31,60^ (Supplementary Fig. 5a). We discovered that ZMYND11 can physically interact with HNRNPA1 via its MYND domain, and this interaction is crucial for ZMYND11 to counteract HNRNPA1-induced aggressive phenotypes in prostate cancer cells (Figs. 3, 4 and Supplementary Figs. 3, 4). Consequently, we aimed to ascertain whether ZMYND11 influences the alternative splicing of PKM pre-mRNA and affects the function of cancer cells. Notably, full-length ZMYND11, but not the MYND-domain-deleted variant, inhibited PKM2 and promoted PKM1 isoform formation at both the mRNA (Fig. 5a) and protein levels (Fig. 5b). Conversely, ZMYND11 knockdown via shRNA decreased PKM1 and increased PKM2 isoform formation (Fig. 5c, d). Consistently, full-length ZMYND11 obstructed the stimulatory effects of HNRNPA1 on PKM2 formation (Fig. 5e, f), further demonstrating the inhibitory effect on HNRNPA1-mediated PKM splicing. PKM2, a splicing variant of pyruvate kinase, is a vital determinant of the cancer metabolism phenotype and selectively enhances proliferative capability for tumor cell growth in vivo.^61,62^ Therefore, we investigated whether ZMYND11 mitigates the aggressive phenotype of prostate cancer cells induced by PKM2 overexpression. The results indicated that full-length ZMYND11, unlike the MYND-domain-deleted variant, countered the enhancement of cell growth, migration, and invasion induced by PKM2 overexpression (Fig. 5g–j and Supplementary Fig. 5b–e).

The Warburg effect, characterized by increased glucose uptake and lactate production, offers a selective advantage for cancer tumorigenesis and progression. The PKM2 isoform plays a crucial role in enhancing the Warburg effect in cancer cells.^61,63–65^ To investigate whether ZMYND11 also influences the Warburg effect, we assessed lactate production and glucose uptake in cells. Full-length ZMYND11, but not the MYND-domain-deleted mutant, significantly reduced lactate production and glucose uptake (Fig. 5k and Supplementary Fig. 5f), whereas silencing ZMYND11 led to an increase in both parameters (Fig. 5l and Supplementary Fig. 5g). Additionally, we analyzed RNA splicing in the ZMYND11 knockdown prostate cell line 22Rv1. The PKM2 isoform was elevated following ZMYND11 knockdown (Fig. 5m). An increase in the PKM2 isoform was also observed in a cohort of 134 human prostate tumors compared to paired normal tissue samples^59^ (Fig. 5n–p). Collectively, these findings demonstrate that ZMYND11 suppresses aerobic glycolysis in prostate cancer cells by inhibiting HNRNPA1-dependent PKM splicing.

### ZMYND11 specifically binds the 194 arginine residues within the RGG motif of HNRNPA1

**Fig. 6:**
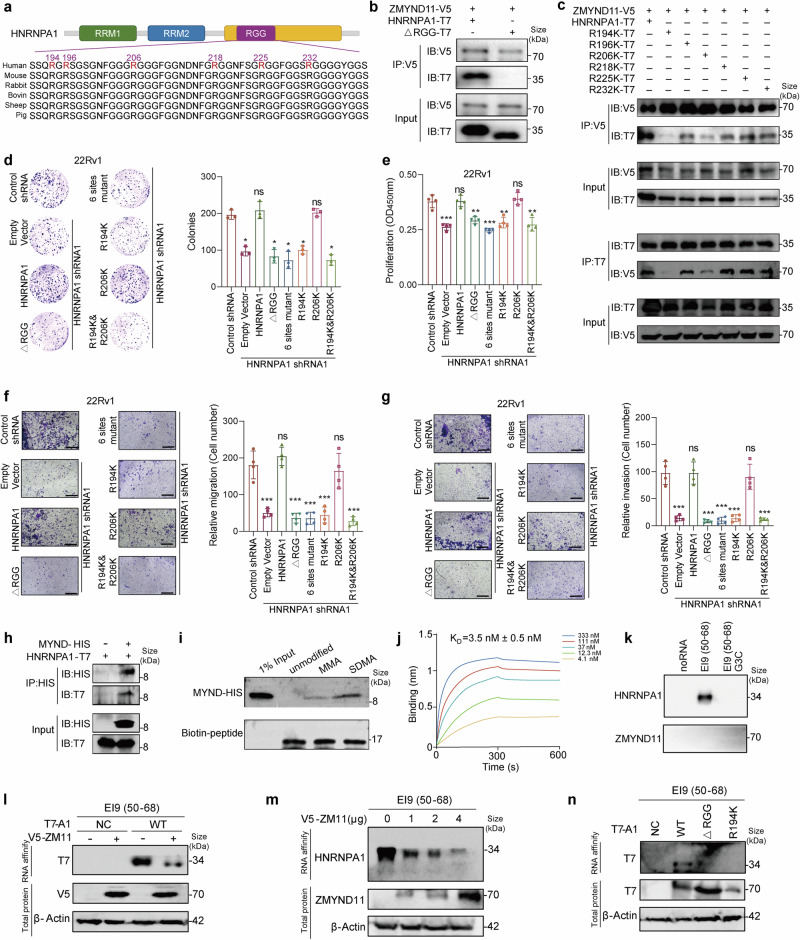
HNRNPA1 arginine 194 methylation is required for its oncogenic function and recognition by ZMYND11. **a** Schematic diagram showing the protein domain organization of HNRNPA1. Shown are amino acid sequence conservation of the glycine-arginine-rich (RGG) motif with highlighted arginine (R) residues at the indicated position. RRM: RNA recognition motif. **b** Whole-cell lysates from HEK293T cells expressing V5-tagged ZMYND11 and T7-tagged HNRNPA1 or its mutant lacking the RGG-box were subjected to co-immunoprecipitation (co-IP) with anti-V5 antibody followed by immunoblotting. **c** The lysates from HEK293T cells expressing V5-tagged ZMYND11 and T7-tagged HNRNPA1 or the indicated mutant constructs, were immunoprecipitated with anti-V5 antibody followed by immunoblotting, and the reciprocal co-IP shown in the bottom. Representative images and quantification of colony-formation (**d**), cell proliferation (**e**), migration (**f**), and invasion (**g**) of 22Rv1 cells with stable knockdown of HNRNPA1 and rescue by co-transfection with the indicated constructs. Scale bar, 400 μm. Statistical significance was assessed using two-tailed Student’s *t* test. ns: not significant, *: *P* < 0.05, **: *P* < 0.01, ***: *P* < 0.001. **h** His-tag pull-down assay investigating direct interactions between the MYND domain of ZMYND11 and HNRNPA1. **i** Pull-down assays between the MYND domain of ZMYND11 and the biotin-labeled peptides carrying R194 monomethylation (MMA) or symmetric demethylation (SDMA). **j** Bio-layer interferometry (BLI) binding assay of ZMYND11-MYND and HNRNPA1-SDMA-R194. **k** The 22Rv1 nuclear extract (NE) were affinity-purified using the indicated biotin-labeled RNAs, and the eluted proteins were detected using anti-HNRNPA1 and ZMYND11 antibodies. **l** 22Rv1 cells were co-transfected with ZMYND11-V5 and HNRNPA1-T7 plasmid and RNA affinity purification was performed as in a using biotin-labeled RNA E19 (50–68). **m** 22Rv1 cells were transfected with ZMYND11-V5 plasmid at the indicated doses and RNA affinity purification was performed as in (**k**) using biotin-labeled RNA E19. **n** 22Rv1 cells were transfected with the indicated HNRNPA1-truncated constructs or HNRNPA1 R194K mutant and RNA affinity purification was performed as in (**k**) using biotin-labeled RNA E19 (50–68)

To delve deeper into the molecular and biochemical basis of the specific interaction between ZMYND11 and HNRNPA1, we focused on the structural features of HNRNPA1. HNRNPA1 comprises two RRMs and a glycine-rich Arg-Gly-Gly (RGG) domain at the carboxyl (C)-terminus (Fig. 6a), which are involved in RNA binding and protein interactions, respectively.^29^ Mass spectrometry analysis of immunopurified HNRNPA1 identified diverse posttranslational modifications, notably frequent arginine methylation within the RGG-motifs (Supplementary Fig. 6a). Arginine methylation in RGG motifs is known to influence RNA binding,^66^ stress granule localization, and formation,^23,67,68^ yet its role in protein-protein interactions and recognition is less understood. Given that ZMYND11 is recognized as an epigenetic reader that specifically recognizes H3.3K36me3 through its PWWP domain,^3,4^ is dysregulated in cancers including prostate cancer, potentially restricting HNRNPA1-mediated stress granule formation and oncogenic activity, we hypothesized that ZMYND11 might also function as a nonhistone reader, recognizing methylated HNRNPA1 through its MYND reader module. To test this, we examined whether the RGG domain of HNRNPA1 is critical for its interaction with ZMYND11. We accordingly created an HNRNPA1 mutation construct lacking the RGG-domain and co-expressed it with V5-tagged ZMYND11 in HEK293T cells. This experiment revealed that the deletion of the RGG domain significantly reduced the binding of ZMYND11 to HNRNPA1 (Fig. 6b), indicating the essential role of the RGG domain in mediating this interaction. Considering that six arginine residues within the RGG motif are potential methylation sites,^69^ we further explored which methylation sites contribute to ZMYND11 interaction by generating single-site mutants by replacing arginine (R) with lysine (K), including R194K, R196K, R206K, R218K, R225K, and R232K, and individually co-expressing them with ZMYND11. The results showed that the R194K mutation notably diminished HNRNPA1 binding to ZMYND11 (Fig. 6c and Supplementary Fig. 6b-d), highlighting the critical role of the R194 residue in this protein-protein interaction. Furthermore, functional assays demonstrated that the R194K mutant, unlike the R206K mutant, significantly reduced colony formation, cell growth, migration, and invasion (Fig. 6d–g and Supplementary Fig. 6e), despite the latter also impacting the HNRNPA1-ZMYND11 interaction. This suggests the indispensability of the R194 residue for tumor-promoting function of HNRNPA1. Altogether, these findings indicate that ZMYND11 specifically binds to the arginine residue at position 194 in the RGG motif of HNRNPA1.

### ZMYND11 specifically recognizes symmetric dimethyl arginine (SDMA) of HNRNPA1

Arginine methylation is a post-translational modification present in a wide range of nuclear and cytosolic proteins, including histones, signaling molecules, and RNA splicing factors.^70^ This methylation on arginine residues can occur in three forms: ω-NG-monomethyl-arginine (MMA), ω-NG,NG-asymmetric dimethyl arginine (ADMA), and ω-NG,NG-symmetric dimethyl arginine (SDMA).^71^To determine the specific arginine methylation on the R194 residue, we overexpressed T7-tagged wild-type HNRNPA1 alongside six arginine-mutated HNRNPA1 variants in 22Rv1 cells. Immunoprecipitation (IP) of T7-tagged proteins revealed no differences in MMA, ADMA, or SDMA levels between the wild-type and the six single-arginine-mutated HNRNPA1 variants (Supplementary Fig. 6f), indicating that a single residue mutation does not alter methylation status. Consequently, we created mutants with all six arginine sites of HNRNPA1 and an unmutated R194 residue for another independent IP assay. This revealed that the R194 residue undergoes MMA and SDMA modifications (Supplementary Fig. 6g). To further investigate the binding affinity of ZMYND11 for arginine-methylated HNRNPA1, we purified the recombinant MYND domain protein of ZMYND11. Co-immunoprecipitation experiments demonstrated that this purified fragment could directly bind to ectopically expressed HNRNPA1 (Fig. 6h). A subsequent pull-down assay confirmed that the MYND domain of ZMYND11 specifically recognizes the SDMA modification of HNRNPA1 at the R194 site (Fig. 6i). We further identified the interaction between ZMYND11-MYND and HNRNPA1-SDMA-R194 using bio-layer interferometry. The BLI analysis showed that ZMYND11 specifically recognizes the SDMA modification of HNRNPA1 with a KD value of 3.5 nM (Fig. 6j). Altogether, these findings demonstrate that ZMYND11 can specifically identify the SDMA modification of HNRNPA1.

### ZMYND11 blocks the binding of HNRNPA1 to intronic sequences flanking PKM Exon 9

Previous studies have shown that the HNRNPA1 protein inhibits the inclusion of PKM Exon 9 (E9) by binding to the intronic UAGGGC sequences flanking exon 9 (EI9), thereby facilitating the formation of the PKM2 isoform.^30^ To explore the impact of ZMYND11 on the binding ability of HNRNPA1 to PKM E9, we conducted RNA affinity chromatography using biotin-labeled RNA corresponding to EI9 (50–68) containing a UAGGGC sequence, EI9 (50–68) G3C (mutation of the G3 nucleotide to C in UAGGGC), or EI9 (1–20) (negative control), as previously described (see also Methods).^72^

Strong binding was observed between HNRNPA1 and the EI9 (50–68) sequence of PKM, whereas mutating the G3 nucleotide of EI9 (50–68) to C resulted in no binding to HNRNPA1. We found that ZMYND11 did not directly bind to the mRNA sequences of PKM (Fig. 6k), indicating that ZMYND11 binds to HNRNPA1 in an RNA-independent manner. When ZMYND11 was overexpressed, the binding of HNRNPA1 to EI9 (50–68) of PKM was completely blocked (Fig. 6l). Additionally, ZMYND11 blocked the binding of HNRNPA1 to EI9 (50–68) of PKM in a dose-dependent manner (Fig. 6m).

To investigate which domain of HNRNPA1 binds to EI9 (50–68) of PKM, T7-tagged HNRNPA1 and its truncated constructs were ectopically expressed in 22Rv1 cells, and RNA affinity chromatography was performed using biotin-labeled RNA corresponding to EI9 (50–68). Only constructs containing the arginine methylation of HNRNPA1 at the R194 site, rather than other regions of HNRNPA1, bound to the EI9 sequence of PKM, suggesting that the arginine methylation on the R194 residue of HNRNPA1 is essential for binding to EI9 sequences of PKM (Fig. 6n).

Taken together, our results indicate that ZMYND11 blocks the binding of HNRNPA1 to PKM mRNA by competitively binding to the arginine residues at the R194 site of HNRNPA1.

### ZMYND11 specifically recognizes SDMA of HNRNPA1 through the 572 tyrosine residue

**Fig. 7:**
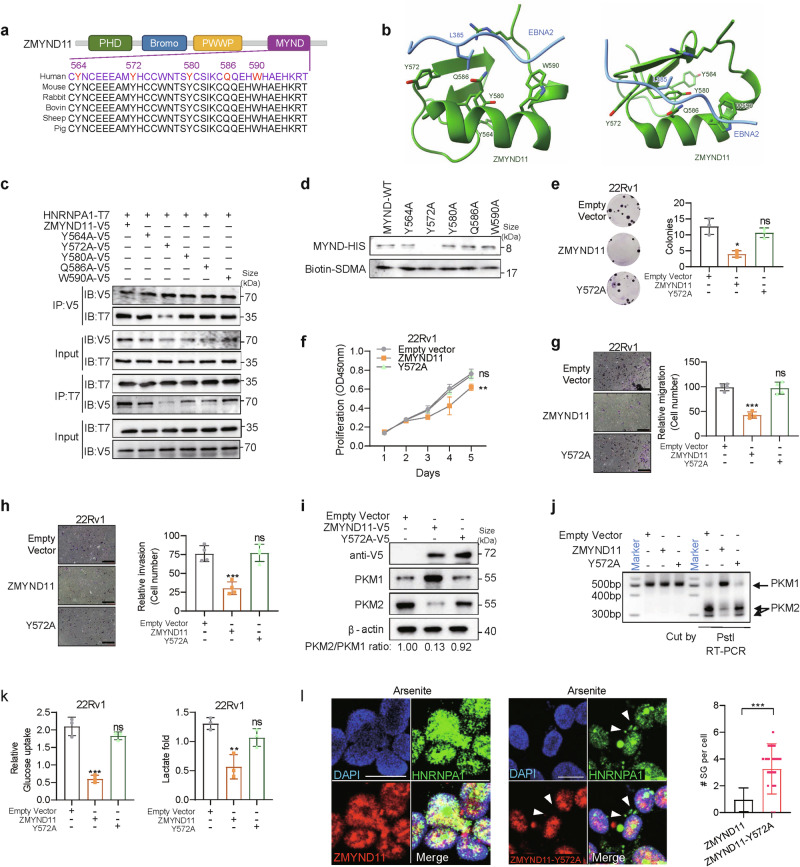
Tyrosine residue 572 of ZMYND11 is functionally important and essential for specifically reading SDMA on HNRNPA1. **a** Schematic representation of the MYND domain of ZMYND11. Shown is MYND domain organization along with potential candidate amino acid residues likely to be involved in binding with HNRNPA1. MYND domain conserved across species is shown. **b** Two views of the crystal structure (PDB:5HDA) for the MYND domain of ZMYND11 complexes with EBNA2 (blue). Potential candidate amino acid residues responsible for recognition with HNRNPA1 are show in sticks. **c** Co-immunoprecipitation assay in HEK293T cells expressing T7-tagged HNRNPA1 and V5-tagged ZMYND11 or the indicated mutants. **d** Pull-down assays demonstrated protein-protein interactions between HIS-tagged MYND domain of ZMYND11 or the mutants and the biotinylated peptide containing R194 SDMA. Representation and quantification of colony-forming (**e**), cell proliferation (**f**), migration (**g**), and invasion (**h**) of 22Rv1 cells overexpressing ZMYND11 or the Y572A mutant. Scale bar, 400 μm. *P < 0.05, **P < 0.01, ***P < 0.001, were examined by two-tailed Student’s *t* test. PKM splicing assay (**i**) and immunoblots for the indicated PKM isoforms (**j**) were performed in 22Rv1 cells ectopically expressing ZMYND11 or the Y572A mutant. **k** Relative glucose uptake and lactate production were measured in 22Rv1 cells with an ectopical expression of ZMYND11 or Y572A mutant. ns, not significant, *P < 0.05, **P < 0.01, ***P < 0.001, two-tailed Student’s *t* test. **l** Fluorescence colocalization microscopy analysis of ZMYND11 or the Y572A mutant with HNRNPA1 in 22Rv1 cells treated with sodium arsenite or control. Note that stress-induced cytoplasmic accumulation of HNRNPA1 was substantially attenuated by wild-type ZMYND11, but not the Y572A mutant. Arrows, stress granules in cells. Mean ± SD, was assessed using two-tailed Student’s *t* test. *** represents *p* < 0.005. Scale bars, 25 μm

To elucidate the molecular mechanism by which the ZMYND11 MYND domain recognizes HNRNPA1, we analyzed the crystal structure of the ZMYND11-MYND domain in complex with the Epstein-Barr virus nuclear antigen 2 (EBNA2) (PDB:5HDA) to identify specific amino acid residues in ZMYND11 responsible for binding to HNRNPA1.^73^ This analysis identified five residues within the MYND domain of ZMYND11: Y564, Y572, Y580, Q586 and W590, which are crucial for binding to EBNA2 and could potentially contribute to the recognition of HNRNPA1 (Fig. 7a, b). Mutagenesis assays involving the substitution of these residues with alanine (A) revealed that the Y572A mutation in ZMYND11 significantly reduced its interaction with HNRNPA1 (Fig. 7c and Supplementary Fig. 7a), highlighting the critical role of the Y572 residue in this interaction. This finding was corroborated by two independent pull-down assays, confirming that the ZMYND11 Y572A mutant protein failed to recognize the SDMA modifications on HNRNPA1 (Fig. 7d and Supplementary Fig. 7b). Additionally, the Y572 mutant negated the inhibitory effects of WT ZMYND11 on colony formation, cell growth, migration, and invasion in prostate cancer cells, indicating the essential role of the Y572 residue in mediating the tumor-suppressive function of ZMYND11 (Fig. 7e-h). As expected, WT ZMYND11, but not the Y572A mutant, inhibited PKM2 and promoted PKM1 isoform formation at both protein (Fig. 7i) and mRNA levels (Fig. 7j). The Y572A mutant did not affect lactate production and glucose uptake (Fig. 7k and Supplementary Fig. 7c). Furthermore, immunofluorescence analysis showed that WT ZMYND11, unlike the Y572A mutant, significantly reduced the stress-induced cytoplasmic accumulation of HNRNPA1 in response to SA treatment (Fig. 7l). Altogether, these results demonstrate that ZMYND11 specifically recognizes the SDMA modification of HNRNPA1 through the Y572 residue, which is crucial for regulating PKM splicing and preventing the cytoplasmic relocalization of HNRNPA1 under stress.

### Depletion of PRMT5 impairs the interaction between ZMYND11 and HNRNPA1

**Fig. 8:**
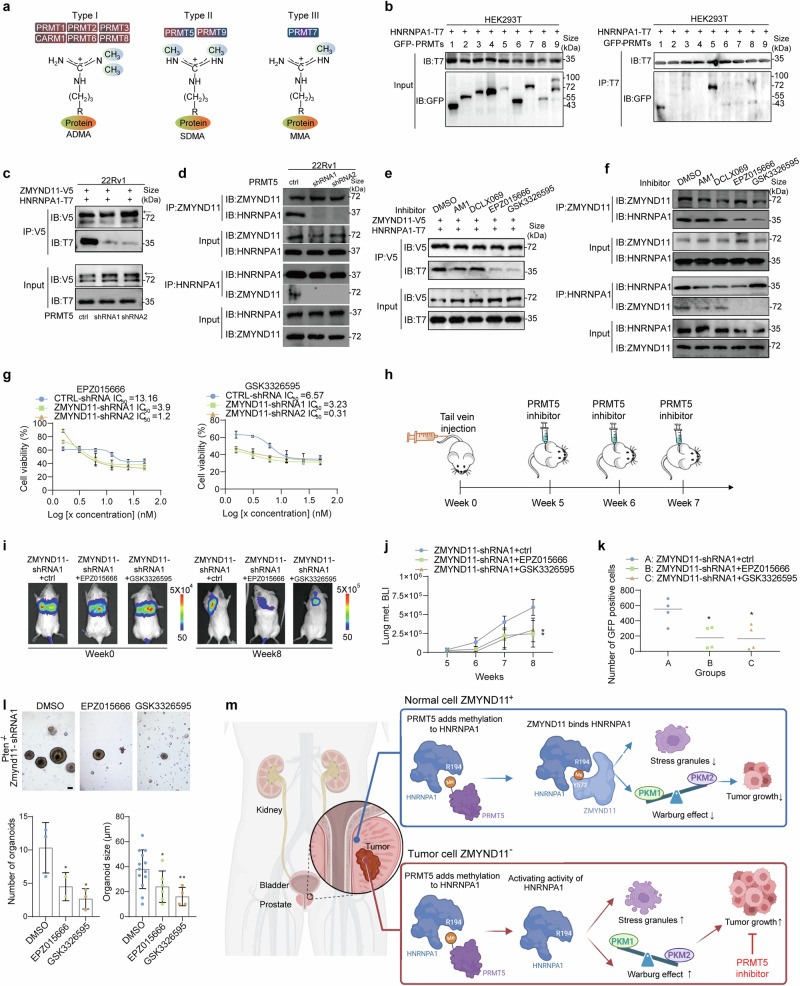
Pharmacological inhibition of PRMT5 impairs ZMYND11-HNRNPA1 interaction and suppresses in vivo metastatic capacity of prostate cancer cells with ZMYND11-low expression. **a** Schematics of arginine methylation states by PRMT family of enzymes. **b** Co-immunoprecipitation assay of HEK293T cells expressing T7-tagged HNRNPA1 and the indicated GFP-tagged PRMT family member. **c** Co-immunoprecipitation assays using 22Rv1 cell protein extracts co-expressing V5-tagged ZMYND11 and T7-tagged HNRNPA1 while having stable expression of control or shRNAs against PRMT5. **d** Immunoprecipitation-Western blot analysis of an endogenous interaction between ZMYND11 and HNRNPA1 in 22Rv1 cells stably expressing control or ZMYND11-targeting shRNAs. **e** Immunoprecipitation using V5-tag antibodies from 22Rv1 cells ectopically co-expressing ZMYND11 and HNRNPA1 while treated with PRMT1 inhibitors (AM1 or DCLX069, 50 μM, 48 h) or PRMT5 inhibitors (EPZ015666 or GSK3326595, 10 μM, 48 h). **f** Co-immunoprecipitation assay of an endogenous interaction between ZMYND11 and HNRNPA1 in 22Rv1 cells treated with the indicated PRMT1/5 inhibitor. **g** Half-inhibitory concentration (IC50) test of two PRMT5 inhibitors (EPZ015666 or GSK3326595, 10 μM, 48 h) for ZMYND11 knockdown or control cells in 22Rv1. **h** Schematics of in vivo mouse experiments with PRMT5-Selective Inhibitors. 22Rv1 cells with stable knockdown of ZMYND11 while expressing luciferase were injected into the tail-vein of male NOD-SCID mice. At 5, 6, and 7 weeks after injection, mice were subject to intraperitoneal treatment with the indicated PRMT5 inhibitors. Shown are the representative images at indicated time points (**i**) and weekly quantification of BLI photon flux of lung metastasis in mice (**j**). Errors bar, ±SD, *n* = 5. *p < 0.05, **p < 0.01, ***p < 0.001, Student’s *t* tests. **k** FACS-analysis of GFP-positive 22Rv1 cells (CTCs) in the peripheral blood of SCID mice (*n* = 4). The scatter plot indicated the number of CTCs recovered from each mouse treated with vesicles or PRMT5 inhibitors. Statistical significance was assessed using two-tailed Student’s *t* test. * *p* < 0.05. **l** Organoid images derived from prostatic ZMYND11 knockdown *Pten*^*−/−*^ mice treated with PRMT5 inhibitors (EPZ015666 or GSK3326595, 10 μM); quantitative results are representative of 3 experiments shown at the right. Scale bar: 40 μm. Errors bar, ±SD, *n* = 5. *p < 0.05, **p < 0.01, Student’s *t* tests. **m** A model illustrating how ZMYND11 recognition of arginine methylation constrains HNRNPA1-mediated tumor progression is proposed. Top: In normal cells with higher expression of ZMYND11, the protein can specifically recognize the R194 methylation of HNRNPA1, which is catalyzed by PRMT5. This interaction inhibits HNRNPA1’s involvement in stress granule formation and the alternative splicing of PKM, thereby mitigating tumor aggressiveness. Bottom: In tumor contexts with reduced ZMYND11 expression, HNRNPA1 promotes the formation of stress granules and shifts the PKM isoform balance towards a higher PKM2/PKM1 ratio, contributing to tumor progression. In this scenario, PRMT5 inhibitors emerge as potential therapeutic agents capable of curtailing cancer progression by disrupting the methylation-dependent interaction between ZMYND11 and HNRNPA1, ultimately impairing HNRNPA1’s tumor-promoting activities

Arginine residue methylation is facilitated by the arginine methyltransferase (PRMT) family, categorized into type I, II, and III based on their methyl-arginine products. Type I PRMTs, including PRMT1, 2, 3, 4, 6, and 8, catalyze the formation of monomethylarginine (MMA) and asymmetric dimethylarginine (ADMA),^34^ while PRMT5 and 9, type II PRMTs, catalyze MMA and SDMA. PRMT7, a type III PRMT, exclusively catalyzes MMA (Fig. 8a). Our findings indicated that the methylation of the R194 residue in HNRNPA1 predominantly results in SDMA (Fig. 6I). To identify which PRMTs are involved in HNRNPA1 methylation, we conducted a Co-IP assay by co-expressing T7-tagged HNRNPA1 with nine GFP-tagged PRMTs in HEK293T cells, revealing that PRMT1 and PRMT5 exhibit higher binding affinity towards HNRNPA1 (Fig. 8b). Consistent with previous reports,^55^ PRMT1, PRMT5 and PRMT7 are known to regulate the arginine methylation of HNRNPA1. We then established cell models with knockdown of PRMT1, PRMT5 and PRMT7 individually to examine the impact of PRMT-mediated HNRNPA1 arginine methylation on its association with ZMYND11 (Supplementary Fig. 8a). The results demonstrated that PRMT5 knockdown significantly impaired the interaction between HNRNPA1 and ZMYND11, while the interactions remained unaffected in cells with PRMT1 or PRMT7 knockdown (Fig. 8c and Supplementary Fig. 8b). Moreover, we observed a disruption in the interaction between endogenously expressed ZMYND11 and HNRNPA1 in 22Rv1 or DU145 cells with stable PRMT5 knockdown (Fig. 8d and Supplementary Fig. 8c). Corroborating these findings, the protein-protein interaction between HNRNPA1 and ZMYND11 was significantly reduced upon treatment with PRMT5 inhibitors (EPZ015666 or GSK3326595), while the inhibitory effects were notably weaker for PRMT1 inhibitors AM1 and DCLX069 (Fig. 8e, f). Overall, our results underscore that ZMYND11 specifically recognizes SDMA in HNRNPA1, with PRMT5 being crucial for the arginine methylation and essential for the interaction between ZMYND11 and HNRNPA1.

### Pharmacological inhibition of PRMT5 suppresses the growth of tumors with low ZMYND11 expression

Considering the pivotal role and clinical relevance of PRMT5 inhibitors in cancer progression,^37,38^ we explored whether PRMT5 inhibitors could serve as an effective therapeutic strategy for prostate cancer characterized by low ZMYND11 expression. As anticipated, PRMT5 inhibitors (EPZ015666 and GSK3326595) significantly reduced cell growth, colony formation, migration, and invasion in ZMYND11 knockdown cells, whereas the inhibitors did not significantly affect control cells (Supplementary Fig. 8d-f). The half-inhibitory concentration (IC50) test further confirmed that these inhibitors were more effective in eliminating ZMYND11 knockdown cells compared to control cells (Fig. 8g). Remarkably, in a bioluminescent imaging (BLI)-based lung metastasis mouse model, established by tail vein injection of prostate cancer cells with stable ZMYND11 knockdown, treatment with PRMT5 inhibitors EPZ015666 or GSK3326595 substantially reduced lung metastasis (Fig. 8h-j). Additionally, GFP-positive circulating cancer cells in the blood were notably decreased following treatment with these inhibitors (Fig. 8k). Consistent with the observed in vivo phenotype, PRMT5 inhibitors (EPZ015666 and GSK3326595) markedly inhibited organoid formation (Fig. 8l). These findings suggest that pharmacological inhibition of PRMT5 effectively curbs the growth of prostate cancer cells and tumors, positioning ZMYND11 as a potential predictive biomarker for improving the clinical management of patients.

## Discussion

ZMYND11 has traditionally been recognized as an epigenetic reader that specifically binds to the histone mark H3.3K36me3, modulating RNA Pol II during the elongation phase to suppress gene expression.^4,5^ In this study, we reveal a novel and independent role for ZMYND11 in regulating HNRNPA1 and its downstream networks through PRMT5-induced arginine methylation. This noncanonical function of ZMYND11 furthers our understanding of its tumor-suppressive mechanisms and elucidates its interactions with oncogenic pathways, such as the HNRNPA1-PKM2 axis. We propose a model in which ZMYND11, through its MYND domain—rather than the conventional PBP ‘epigenetic reader’ modules—recognizes the methyl-modified nonhistone protein HNRNPA1. Notably, the Y572 site within the MYND domain is pivotal for this interaction. These findings enhance our understanding of ZMYND11’s role as a tumor suppressor, highlighting its previously unrecognized function as a nonhistone methylation reader (Fig. 8m).

HNRNPA1 is known to play a crucial role in increasing the PKM2/PKM1 ratio through alternative splicing, thereby enhancing aerobic glycolysis. This increase in HNRNPA1 expression is driven by the oncogenic transcription factor c-Myc.^30^ Despite the substantial overexpression of MYC, HNRNPA1, and PKM2 in most tumors,^30,74,75^ it is important to note that the MYC-HNRNPA1-PKM2 axis is not universally responsible for tumorigenesis and tumor progression across all cancer types.^76^ This observation suggests the existence of regulatory mechanisms that can modulate or inhibit the activity of this axis. Herein we uncovered a novel, noncanonical role of the epigenetic reader ZMYND11 as a nonhistone methyl reader, specifically recognizing the methylation of arginine 194 (R194) on HNRNPA1. This recognition downregulates the oncogenic functions of HNRNPA1, particularly its role in PKM splicing and aerobic glycolysis (as illustrated in Fig. 8m). Through a series of functional assays, we demonstrated that the R194 mutant of HNRNPA1 significantly impairs its oncogenic potential, leading to reduced colony formation, cell proliferation, migration, and invasion. Interestingly, while the R206 mutant still disrupts the interaction between ZMYND11 and HNRNPA1, it does not exhibit the same inhibitory effects on HNRNPA1’s oncogenic activities. This observation raises intriguing questions about the distinct biological roles of these two arginine residues. The fact that the R206 mutant does not mirror the effects of the R194 mutant, despite disrupting the ZMYND11-HNRNPA1 interaction, suggests that R206 might be involved in alternative regulatory pathways or mechanisms independent of ZMYND11. Future research should focus on elucidating the specific functions of the R206 site, as this could uncover additional layers of regulation and potential therapeutic targets.

Our findings clearly indicate that ZMYND11 plays a critical role in inhibiting HNRNPA1-mediated oncogenic activities, including PKM splicing and stress granule formation, both of which are crucial for cancer cell survival and proliferation. Clinically, we observed a strong inverse correlation between ZMYND11 expression and key indicators of tumor aggressiveness, such as metastasis, advanced tumor stages, and poor patient prognosis. These results position ZMYND11 as a promising biomarker for the diagnosis and prognosis of certain cancers and underscore its potential as a target for therapeutic intervention.

HNRNPA1 proteins are integral to the assembly of stress granules through liquid-liquid phase separation,^26^ a process vital for cellular compartmentalization and response to stress. As an RBP, HNRNPA1 harbors two RRM and a glycine-rich domain, which includes multiple Arg–Gly–Gly (RGG) tripeptide repeats. These repeats are crucial for mediating protein-protein interactions, RNA-binding, and cellular compartmentalization.^29–31^ In normal cells, SG formation serves a protective role by safeguarding cells from stress-induced damage. However, in cancer cells, SGs can contribute to tumor survival and immune evasion, thereby promoting tumorigenesis.^53^

The mechanisms underlying SG formation are not fully understood, and further elucidation is critical for comprehending their role in cancer. In this study, we discovered a novel mechanism by which the MYND domain of ZMYND11 specifically recognizes the symmetrically dimethylated RGG motif of HNRNPA1. This interaction limits the cytoplasmic translocation of HNRNPA1, thereby reducing SG formation (Fig. 8m). The reduction of SGs in cancer cells is particularly significant, as these structures are known to decrease apoptosis and promote cell survival under stress conditions. Our apoptosis assays further confirmed that ZMYND11 induces both early and late apoptosis in prostate cancer cells under stress, suggesting its role as a potential tumor suppressor. However, while our study highlights the importance of ZMYND11 in modulating SG formation and promoting apoptosis, the direct mechanisms by which ZMYND11 induces apoptosis remain unclear and warrant further investigation. Understanding how ZMYND11 triggers apoptosis could provide deeper insights into its tumor-suppressive functions and offer new therapeutic avenues for targeting SGs and enhancing cancer cell vulnerability to stress-induced apoptosis.

Cancer cells can actively remodel the tumor microenvironment by secreting metabolites, thereby creating an immunosuppressive environment conducive to tumor growth and metastasis.^77^ The tumor microenvironment often includes a range of stress conditions, such as hypoxia, oxidative stress, metabolic stress, and endoplasmic reticulum (ER) stress, all of which arise due to the high metabolic demands of proliferating cancer cells and the limited oxygen and nutrient supply from surrounding vasculature.^1^ Chemotherapeutic agents, which target critical components like DNA, RNA, and proteins in cancer cells, have been shown to disrupt the recruitment of SG components, thereby inhibiting cancer progression and enhancing tumor clearance.^78^ Targeting SGs thus represents a promising strategy for cancer therapy. However, chemoresistance remains a major challenge for effective cancer treatment, often leading to poor prognosis. Recent studies suggest that various chemotherapeutic drugs, including 5-Fluorouracil, arsenic trioxide, and paclitaxel, can trigger SG assembly, which may contribute to the development of chemotherapy resistance.^50–52^ This observation highlights the potential of targeting HNRNPA1 or ZMYND11 to overcome resistance to cancer therapies.

Our research has demonstrated that SDMA modifications on the RGG domains of HNRNPA1 are catalyzed by PRMT5. PRMT5 inhibitors are currently being explored as a potential cancer treatment, with various mechanisms proposed to explain their effectiveness.^35,37,38,40,79^ Our findings indicate that PRMT5 inhibitors can effectively suppress cell growth in ZMYND11-low-expressing cells in vitro and inhibit tumor progression in vivo. This suggests that ZMYND11 could serve as a valuable biomarker for guiding clinical diagnostics and therapy in cancer, particularly when considering the use of PRMT5 inhibitors. Moreover, with ongoing clinical trials of a PRMT5 inhibitor (NCT03614728), our results suggest that assessing ZMYND11 expression levels could enhance the therapeutic potential of PRMT5 inhibition in cancer patients with lower ZMYND11 expression. Future studies should aim to include clinical samples collected before and after PRMT5 inhibitor treatment to rigorously test this hypothesis in well-defined cohorts of cancer patients.

In summary, this study is the first to reveal the non-canonical function of the epigenetic reader ZMYND11 in recognizing nonhistone methylation. Our findings demonstrate that ZMYND11 exerts a dual tumor-suppressive role: first, by inhibiting HNRNPA1-mediated stress granule formation, ZMYND11 promotes apoptosis in cancer cells under stress; second, ZMYND11 suppresses aerobic glycolysis by inhibiting HNRNPA1-driven alternative splicing of PKM2. This dual functionality highlights ZMYND11 as a key regulator of cellular stress responses and metabolic reprogramming in cancer cells. Furthermore, our study suggests that disrupting the methylation-dependent interaction between ZMYND11 and HNRNPA1 with PRMT5 inhibitors offers a promising therapeutic strategy. By targeting this interaction, PRMT5 inhibitors could effectively impair HNRNPA1’s tumor-promoting activities, thereby curbing cancer progression and offering new avenues for cancer treatment.

## Materials and methods

### Mouse experiments

The animal studies described were conducted in accordance with the guidelines of the Animal Care and Use Committee of the School of Basic Medical Sciences at Shanghai Medical College of Fudan University, under approval number 20220228-014. These protocols strictly adhered to the National Institutes of Health Guide for the Care and Use of Laboratory Animals, ensuring the ethical and humane treatment of all animals involved in our research.

This study details a subcutaneous tumor model where 1×10^7^ 22Rv1 or 5×10^6^ DU145 cells in 100 μL of PBS were injected into the left flanks of 5-week-old male BALB/c nude mice. Six weeks post-injection, the mice were euthanized, and tumor volumes were calculated. In the spontaneous lung metastasis model, 2×10^6^ 22Rv1 or 1×10^6^ DU145 cells in 100 μL of PBS were injected into the tail vein of 5-week-old male NOD-SCID mice. D-Luciferin from Yeasen (40901ES) was dissolve in PBS to make a 15 mg/ml (1 g in 66.7 ml PBS), and 100 uL/mouse (1.5 mg/mouse) was administered via intraperitoneal injection to assess lung metastasis measured by bioluminescent imaging (BLI) using an in vivo imaging system (IVIS). BLI was measured weekly, and blood was collected for FACS analysis at the endpoint of the experiment.

In the orthotopic prostate cancer xenograft mouse model, 8-week-old C57BL/6 J male mice were anesthetized using 1.5–2 Vol% Isoflurane with an oxygen flow of 0.6 l/min in a separate box. The mice were positioned on a heated operating table with the left side upwards. The skin was cleaned, shaved, and sterilized. A cell suspension of 5×10^5^ RM1 cells in 25 μL of PBS mixed with half Matrigel was orthotopically injected into the prostate using an insulin needle. The seminal vesicle and the prostate were carefully repositioned back into the visceral cavity, and the abdominal wall was closed using sutures. After the procedure, the mice were kept warm in a separate box while recovering from anesthesia. After 5 weeks, these mice were sacrificed, and the orthotopic tumors were measured. The mice were randomly assigned to groups based on similar body weights, and no mice were excluded from the analysis except those that died unexpectedly due to unrelated causes.

### Cell Lines

The cell lines including 22Rv1, LNCaP, PC3, A549 were cultured in RPMI1640 (Invitrogen), while DU145, MDA-MB-231 and HEK293T were grown in DMEM (Invitrogen). Cells were kept at a constant temperature of 37°C in a 5% CO_2_ incubator. The base medium was enriched with 10% fetal bovine serum (FBS, Giboc) and 1% penicillin-streptomycin (BI) to provide necessary nutrients and prevent bacterial contamination.

### Human prostate cancer tissues and non-cancer tissues

The use of human specimens in this study was approved by the Ethics Committee Board at the School of Basic Medical Sciences, Shanghai Medical College of Fudan University (approval number: 2022-Y015). All procedures involving human specimens strictly followed the ethical guidelines of the Declaration of Helsinki. Paraffin-embedded prostate cancer tissues and non-cancer tissues were acquired in according with relevant ethical standards and were approved by the Ethics Committee of Tongji Hospital, Shanghai, China (ID: 2018009). Informed consent was obtained from all participants.

### Quantitative RT-PCR

The RNA was isolated from cultured cell lines or mouse tissues using the RNeasy Mini Kit from QIAGEN, which facilitates the efficient extraction of high-quality RNA. cDNA was synthesized from the isolated RNA using the High-Capacity cDNA Reverse Transcription Kit (TAKARA), containing all necessary reagents and enzymes for efficient reverse transcription. Quantitative RT-PCR analysis employed the SYBR Select Master Mix (TAKARA), inclusive of all components needed for the assay, along with SYBR Green dye for real-time amplification monitoring. Primer sequences for this experiment are detailed in the Supplementary Table 1.

### Western blot

Cells were harvested and lysed with RIPA buffer (50 mM Tris-HCl pH 7.4, 150 mM NaCl, 1 mM EDTA, 1% Triton x-100, 1% Sodium deoxycholate, 0.1% SDS, 1 tablet cocktail protease inhibitors per 10 mL). The lysates were then denatured using pre-boiled 6X SDS sample buffer and boiled for 10 min. The denatured samples were electrophoresed on a polyacrylamide gel, and proteins were transferred onto a nitrocellulose membrane. The membrane was blocked with 5% skim milk and incubated with primary antibodies overnight at 4°C. This was followed by incubation with secondary antibodies conjugated with fluorescent or enzymatic tags. Protein bands were visualized via chemiluminescence or fluorescence, and band intensity was quantified using image analysis software. Nuclear and cytoplasmic fractions were separated using the NE-PER kit (Thermo Fisher Scientific, 78833). The antibodies utilized included Anti-ZMYND11 (CST, 677135), Anti-HNRNPA1 (Proteintech, 67844-1-Ig), Anti-V5 tag (Thermo Fisher Scientific, R96025), HRP-anti-V5 Tag (Thermo Fisher Scientific, R96125), Anti-human GAPDH (Proteintech, 10494-1-AP), HRP-Goat anti-mouse IgG (Proteintech, SA00001-1), HRP-Goat anti-Rabbit IgG (Proteintech, SA00001-2), Anti-Beta Actin (Proteintech, 66009-1-Ig), Anti-PKM1 (Proteintech, 15821-1-AP), Anti-PKM2 (Proteintech, 15822-1-AP), Anti-T7 tag (Bethyl, A190-117A), Anti-PRMT1 (Santa cruz, sc-166963), Anti-PRMT5 (Santa cruz, sc-376937), Anti-PRMT7 (Abclonal, A12159), Anti-G3BP1 (Abclonal, A14836), and Anti-His-Tag (Abclonal, AE003). Detailed information is available at the Supplementary Table 3.

### siRNA transfection

22Rv1 cells at 50%–60% confluence or LNCaP cells at 60%–70% confluence were seeded in 6-well plates. After 24 h, Lipofectamine RNAiMAX reagent (Invitrogen) was mixed with Opti-MEM, and siRNAs were similarly diluted in Opti-MEM. The diluted siRNAs were combined with the Lipofectamine RNAiMAX mixture and incubated at room temperature for 10 min. Subsequently, the siRNA-lipid complexes were added to the cells. The medium was replaced after 24 h, and cells were harvested 48 h post-transfection. The sequences for siRNA are detailed in the Supplementary Table 4.

### Lentiviral constructs, lentivirus production and infection

The shRNA constructs were ordered from Functional Genomics Unit (University of Helsinki) or designed using the BLOCK-iT RNAi Designer (Thermo Fisher Scientific) and inserted into the pLKO.1 Vector (Addgene). The sequences for shRNA are detailed in the Supplementary Table 4. To package third-generation lentiviral vectors, 293 T cells were cultured to 65%-75% confluency in 6-cm dishes. These cells were then trypsinized and seeded into new 6-cm dishes. After 24 h, the growth medium was replaced with fresh, FBS-free DMEM (Invitrogen) supplemented with 0.1% penicillin and streptomycin. The cells were co-transfected with the following plasmids:

- Indicated shRNA construct or overexpression construct (3 μg each)
- pVSVG (envelope plasmid, 1 μg)
- pMDLg/pRRE (packaging plasmid, 1 μg)
- pRSV-Rev (packaging plasmid, 1 μg)

Transfection was performed using 18 μl of PEI (polyethyleneimine). Post- transfection, the medium was replaced with fresh medium, and the virus-containing medium was collected every 12 h, up to six times. This collected lentivirus was filtered through a 0.45 mm filter unit to remove cellular debris, then snap frozen in liquid nitrogen and stored at -80°C for later use. Lentivirus-mediated knockdown involved infecting target cells with lentivirus containing the required shRNA sequence. The virus was removed after 24 h and replaced with normal growth medium containing 1 mg/ml puromycin (Sigma) to eliminate any uninfected control cells. Once control cells were eradicated, successfully infected target cells were maintained in normal growth medium with a reduced puromycin concentration of 0.5 mg/ml to sustain selection pressure. For lentivirus-mediated overexpression, the 22Rv1, LNCaP and DU145 cell lines were generated through lentiviral transduction using vectors cloned into pcDNA3.1 (Addgene) and pCDH (Addgene). Primer sequences for 669 this experiment are detailed in the Supplementary Table 2.

### Co-IP and mass-spectometry (MS) analysis

Cultured cells were rinsed with pre-cooled PBS and lysed using IP buffer (glycerophosphate, 1.5 mM MgCl_2_, and 2 mM ethylenebis (oxyethylenenitrilo) tetraacetic acid (EGTA)). The cell lysates were centrifuged at 10,000 × *g* for 15 min at 4°C to remove intact cells. The supernatant was then incubated overnight with either control immunoglobulin G (IgG) or the primary antibody in IP buffer supplemented with protease inhibitors. This was followed by a 2-h incubation at 4 °C with 20 μL of resuspended volume of Protein G beads (Roche) to pull down bound proteins. The beads were centrifuged at 1000 × *g* for 5 min at 4°C to remove the supernatant and washed four times with IP buffer. The samples were then boiled for 10 min at 95 °C, run on an SDS PAGE gel, and stained with Coomassie Brilliant Blue R-250 (Bio-Rad). The gel bands were excised, destained, trypsinized and subjected to MS analysis for individual protein identitication using liquid chromatography-Tandem Mass Spectrometry (LC-MS/MS) on an Orbitrap Velos Pro mass spectrometer (Thermo Fisher Scientific). The analysis of posttranslational modification employed the same methodology and instrumentation.

### RNA-seq library generation and sequencing

Total RNA from each sample was extracted using various kits such as TRIzol Reagent (Invitrogen), RNeasy Mini Kit (Qiagen), or others. The quantity and quality of the total RNA were assessed using the Agilent 2100 Bioanalyzer (Agilent Technologies, Palo Alto, CA, USA), NanoDrop (Thermo Fisher Scientific Inc.), and 1% agarose gel. For library preparation, 1 μg of total RNA with an RIN value above 6.5 was utilized. Library preparations for next-generation sequencing were constructed following the VAHTS mRNA-seq V3 Library Prep Kit for Illumina (NR611) protocol. Poly(A) mRNA isolation was conducted using either a Poly(A) mRNA Magnetic Isolation Module or an rRNA Removal Kit. mRNA was fragmented and primed using First Strand Synthesis Reaction Buffer and Random Primers, respectively. First-strand cDNA synthesis was carried out using ProtoScript II Reverse Transcriptase, and the second strand was synthesized with Second Strand Synthesis Enzyme Mix. The double-stranded cDNA was purified using beads, then treated with End Prep Enzyme Mix for end repair and dA-tailing in a single reaction, followed by T-A ligation to attach adaptors to both ends. Adaptor-ligated DNA underwent size selection using beads, recovering fragments of approximately 420 bp (insert size of about 300 bp). Samples were PCR amplified for 13 cycles using P5 and P7 primers, where both primers carry sequences for flow cell annealing to facilitate bridge PCR and the P7 primer includes a six-base index for multiplexing. PCR products were cleaned up using beads, validated with a Qsep100 (Bioptic, Taiwan, China), and quantified using a Qubit 3.0 Fluorometer (Invitrogen, Carlsbad, CA, USA). Libraries with distinct indices were multiplexed and loaded onto a NovaSeq6000 instrument following the manufacturer’s instructions (Illumina, San Diego, CA, USA). Sequencing was performed using a 2×150 bp paired-end (PE) configuration. Image analysis and base calling were executed by the NovaSeq6000 Control Software (HCS) + OLB + GAPipeline-1.6 (Illumina) on the NovaSeq6000 instrument.

### RNA-seq data processing and bioinformatics analysis

RNA-seq data quality was assessed using FastQC (version: 0.11.9). Following quality assessment, Low-quality reads were trimmed and adapters were removed with AdapterRemoval (version: 2.3.2).^80^ Cleaned data were then aligned to the reference genome (hg38) using STAR (version: 2.7.9a),^81^ and the aligned BAM files sorted with SAMtools (version: 1.13).^82^ For quantifying alternative splicing (AS) events, we utilized the LeafCutter pipeline, visualizing differential splicing events through the LeafViz application.^83^

### Cell viability and proliferation assays

The resuspended cells were carefully transferred into 96-well cell plate at densities specific to each cell line (4 × 10^3^ for 22Rv1, 2 × 10^3^ for LNCaP, 1 × 10^3^ for DU145 per well, respectively). Cell viability and proliferation were determined using Cell Proliferation Kit II. I, with absorbance read at 450 nm at designated time points as per the kit’s protocol. Data from triplicate wells were statistically analyzed using a two-tailed Student’s t-test.

### Colony formation assays

The resuspended cells after trypsinization were cultured in six-well plates (1 × 10^3^ for 22Rv1 and LNCaP, 200 for DU145 per well, respectively) and allowed to form colonies over 7–14 days. Post-fixation and staining, colonies of at least 50 cells were counted either manually or with image analysis software, and results were expressed as mean colonies ± standard deviation. Statistical significance was determined using a two-tailed Student’s *t* test from triplicate experiments.

### Invasion and migration assays

Cells were detached from the culture dish with trypsin and suspended in serum-free growth medium. The concentration was adjusted to 4 × 10^5^ cells/ml for 22Rv1 or LNCaP, and 2 × 10^5^ cells/ml for DU145. Then, 200 μl of the cell suspension was transferred into 8-mm Transwell inserts, with or without a 100 μl Matrigel coating. The Matrigel was diluted to a concentration of 250 μg/ml with serum-free medium. The lower chambers of the Transwell inserts were filled with 700 μl of standard growth medium. After incubating for 36 h, cells were fixed with 3.7% formaldehyde and permeabilized with methanol, then stained with Wright-Giemsa stain. Cells remaining on the upper surface of the membranes were removed with a cotton swab. The invasive cells that had migrated to the bottom surface of the filters were quantified by counting the number in 12 microscopic fields per membrane at 20× magnification. Statistical analysis was conducted using a two-tailed Student’s *t* test, based on results from three replicate inserts.

### Fluorescence-activated cell sorting (FACS) analysis

The quantification and characterization of circulating tumor cells (CTCs) expressing green fluorescent protein (GFP) in the bloodstream of SCID mice were performed using fluorescence-activated cell sorting (FACS) analysis with a flow cytometer (FACSCelesta, BD, 07060220). Briefly, the blood of SCID mice was treated with red blood cell (RBC) lysis buffer to remove RBCs, followed by centrifugation at 400 *g* for 10 min to pellet the remaining cells. The supernatant was discarded, and the cell pellet was resuspended in 1–2 ml of pre-cooled PBS. The cell suspension was then placed in a 100 mm plate and incubated for 2 days. After incubation, the cells were digested, washed twice with pre-cooled PBS, and subjected to FACS analysis.

### Apoptosis assay

The analysis of apoptosis was conducted using the Annexin V-FITC Apoptosis Detection Kit (Beyotime, C1062M) according to the manufacturer’s protocol. Briefly, cells were treated with SA for 1 h. Both non-adherent and adherent cells were collected through trypsinization and centrifuged at 1000 *g* for 5 min. The cells were then washed three times with ice-cold PBS and re-suspended in 200 μl of binding buffer. Subsequently, 5 μl of Annexin V-FITC and 10 μl of propidium iodide (PI) were added to the cell suspension, which was then mixed for 15 min in the dark. The stained cells were analyzed using a flow cytometer (FACSCelesta, BD, 07060220), and data analysis was performed using FlowJo version 10.

### PKM splicing assays

PKM splicing was performed as previously described.^30^ Total RNA was extracted using TRIzol, and reverse transcription was performed with the PrimeScript RT reagent Kit with gDNAEraser from TAKARA. The cDNA obtained from reverse transcription was then amplified by PCR and digested using PstI. The primer sequences were PKM-F: 5′-CTGAAGGCAGTGATGTGGCC-3′; and PKM-R: 5′-ACCCGGAGGTCCACGTCCTC-3′. Finally, the digested mixtures were resolved by 8% non-denaturing polyacrylamide gel electrophoresis (PAGE).

### Measurement of lactate production

Cells were transfected with specified siRNAs or plasmids for 36 h, followed by incubation in phenol red-free RPMI1640 medium without FBS for 4 h. Lactate accumulation in the medium was measured using the Lactate Colorimetric Assay Kit II (K627-100, BioVision, USA). The background was corrected by subtracting the OD value of fresh phenol red-free RPMI1640 medium. A standard curve of nmol/well based on OD450 nm was established using lactate standard measurement values. Sample OD450 nm values were then plotted on the standard curve to calculate lactate concentrations in the test samples.

### Glucose uptake assay

Cells were transfected with designated siRNAs or plasmids for 36 h, followed by incubation in DMEM medium lacking L-glucose and phenol red for 8 h. The glucose concentration in the media was determined using the Glucose Colorimetric Assay Kit (K606-100, BioVision, USA). Fresh DMEM medium served as the negative control. The experiment was conducted in triplicate to ensure biological replicability.

### Recombinant protein purification

BL21 (DE3) competent *E. coli* cells were utilized for the expression of His-tagged recombinant proteins using pET-28a plasmids. A single colony was grown in LB liquid medium containing 100 μg/ml kanamycin at a volume of 1 L. The culture was agitated at 200 rpm and 37 °C. Upon reaching an OD600 nm of 0.6, indicative of the exponential growth phase, IPTG was added to a final concentration of 1 mM and the culture was incubated at 16 °C for 14 h to induce protein expression. For cell harvesting, the culture was centrifuged at 10,000 *g* for 10 min. The cell pellet was then lysed using a buffer containing 0.3 M NaCl, 50 mM NaH_2_PO_4_ (pH 8.0), and 1 mg/mL lysozyme, followed by sonication for 5 min. The lysate was centrifuged at 10,000 *g* for 30 min, and the clear supernatant was filtered through a 0.45 μm filter. Purification was carried out using a HisSep Ni-NTA 6FF Chromatography Column (20504, Yeasen Biotech), as per the manufacturer’s instructions. The purified protein was dialyzed and washed with imidazole in PBS, then stored at −80 °C for future use.

### Pull-down assay

Biotin-labeled peptides representing R194 monomethylation (MMA), SDMA, or an unmodified fragment were obtained from Nanjing Peptide Biotechnic and individually incubated with a HIS- MYND fusion protein at 4 °C overnight. Following this incubation, the mixture was incubated with Dynabeads (Thermo Fisher Scientific) for an additional 2 h at 4 °C. The beads were then separated using a magnetic frame and washed four times with a buffer containing 0.3% NP-40 in PBS. The samples were then boiled in SDS loading buffer and analyzed via western blotting.

### Bio-layer interferometry (BLI) binding assay

The BLI assay was analyzed as previously mentioned.^84^ Briefly, the binding kinetics of ZMYND11-MYND domain peptides to HNRNPA1 peptides were measured by BLI on an Octet-RED96 (ForteBio). HNRNPA1 peptides carrying symmetric demethylation at arginine 194 (R194 SDMA) on the C-terminal were biotinylated using BirA biotin-protein ligase obtained from a commercial source (NJPeptide, China). Biotinylated R194 SDMA HNRNPA1 peptides were immobilized onto streptavidin-coated biosensors at a concentration of 10 µg/mL. The streptavidin-coated sensors were then incubated with ZMYND11-MYND domain peptides that were serially diluted three-fold, starting from 333 nM, in phosphate-buffered saline with Tween-20 (PBST) for 300 s to allow association. Subsequently, the sensors were immersed in PBST for an additional 300 s to assess dissociation. All binding curves were analyzed using the Data Analysis software 10.0 and fitted to a 1:1 binding model. KD values were calculated based on R2 values greater than a 95% confidence level.

### RNA affinity purification

The RNA affinity purification was performed as previously described.^72^ In brief, each biotin-tagged RNA (1 nmol) was coupled with 100 μL of streptavidin-agarose beads (Sigma, USA) in 500 μL of binding buffer (10 mM Tris-HCl at pH 7.5, 1 mM EDTA, and 2 M NaCl) overnight at 4 °C. Subsequently, the beads were rinsed three times with binding buffer and three times with buffer D (20 mM HEPES at pH 7.9, 20% glycerol, 100 mM KCl, 0.2 mM EDTA, and 0.5 mM dithiothreitol (DTT)). Nuclear proteins from 22Rv1 cells were extracted using a nuclear and cytoplasmic protein extraction kit (P0017S, Beyotime, China). Then, 500 μg of cellular nuclear proteins were incubated with RNA-streptavidin-agarose beads at 30 °C for 30 min. After proteins and RNA binding, the beads were washed three times with buffer D and three times with buffer D lacking glycerol, followed by elution using 1 × SDS. Finally, the elution products were subjected to western blot analysis using antibodies against Flag, T7, ZMYND11, or HNRNPA1.

### Organoid formation assays

Prostate tissues were extracted from *Pten*^*−/−*^ mice to isolate prostate cancer cells, and *Pten*^*−/−*^ organoids were cultured following the protocol in Gao Lab.^85^ The cells were infected with either a control empty vector or *Zmynd11* knockdown lentivirus, followed by selection with puromycin to establish stable cell lines. The cells were enzymatically dissociated and embedded in Matrigel (BD Biosciences) before being cultured in a mouse medium supplemented with 50× diluted B27, 1.25 mM N-acetyl-l-cysteine, 50 ng/ml epidermal growth factor (EGF), 200 nM A83-01, 100 ng/ml Noggin, 500 ng/ml R-spondin 1, 10 μM Y-27632, and 1 nM dihydrotestosterone. For the organoid formation assay, 2000 cells per well were seeded on day 1, and the number and size of the resulting organoids were evaluated on day 14.

### Half maximal inhibitory concentration (IC50) assay

IC50 values for two PRMT5 inhibitors, EPZ015666 and GSK3326595 (both from MCE), were determined at various pre-incubation time points. 22Rv1 cells were plated at 7,000 cells per well in a 96-well cell culture plate and allowed to attach for 24 h. The cells were then treated with the PRMT5 inhibitors EPZ015666 and GSK3326595, and DMSO as a control, across a concentration gradient (1.5625 nM, 3.125 nM, 6.25 nM, 12.5 nM, 25 nM, 50 nM) for 72 h. After the treatment period, the media was removed from the assay plates, and the CCK-8 agent was added, followed by incubation at 37 °C for 4 h. The absorbance of the plates was measured at 450 nm using a spectrophotometer.

### Immunohistochemistry (IHC)

In this research, IHC was conducted utilizing the standard LSAB protocol provided by Dako, based in Carpinteria, CA. Tissue sections were stained for ZMYND11 using a rabbit monoclonal antibody against ZMYND11 (dilution 1:100, sourced from Abcam, USA) and for HNRNPA1 with a rabbit monoclonal antibody against HNRNPA1 (dilution 1:100, obtained from Proteintech, China).

### Immunofluorescence staining and stress granule analysis

Cells were seeded on coverslips in 24-well plates and fixed with 4% paraformaldehyde for 15 min upon reaching 60% confluence. The cell membranes were permeabilized with 0.3% Triton X-100 in PBS at room temperature for 10 min. Subsequently, the coverslips were blocked in 3% BSA in PBS at room temperature for 1 h, followed by incubation with the primary antibody and a fluorescent-labeled secondary antibody overnight at 4 °C or for 1 h at room temperature, respectively. The nuclei were stained with DAPI in mounting buffer (Beyotime Biotechnology, P0131). Imaging was conducted using a Leica TCS SP8 confocal microscope, with image processing and analysis performed using Leica TCS SP8 software.

To induce stress granule formation, cells were treated with sodium arsenite (SA) (0.5 mM, 1 h, Sigma). Stress granules were quantified manually using the G3BP1 marker (Abclonal, A14836, 1:100). For each experimental condition, 50–100 cells were counted, and three images per group were analyzed.

### Clinical analysis

The evaluation of ZMYND11 and HNRNPA1 protein expression in the Fudan cohort was based on IHC staining area and intensity. The staining area was scored as follows: 0 for 0–5%, 1 for 5–25%, 2 for 26–50%, 3 for 51–75%, and 4 for more than 75% staining. Intensity was categorized as 0 for no staining, 1 for weak staining, 2 for moderate staining, and 3 for strong staining. A composite expression score was calculated using the formula CES = 4*(intensity score − 1) + area score, resulting in a range of possible scores from 0 to 12. The Tongji cohort samples were evaluated using the PE Vectra3 scoring software provided by the Olympus VS120 system, which allows for a maximum score of 300 points. The analysis of alternative splicing in the Changhai cohort, which included 134 prostate cancer tissues and matched adjacent normal tissues (NT), was conducted using LeafCutter software.

### Survival analysis

Kaplan-Meier survival analysis was utilized to evaluate the influence of ZMYND11 and HNRNPA1 expression on prostate cancer prognosis across several independent cohorts. Initially, patients were divided into two groups according to the median expression levels of each gene. To explore the synergistic effect of ZMYND11 and HNRNPA1 on patient survival, patients were stratified by the median expression levels of either gene. Subsequently, those with either high ZMYND11 and low HNRNPA1 expression or low ZMYND11 & high HNRNPA1 expression were selected for further analysis. The Kaplan-Meier survival analysis was performed using the “Survival” package (version 3.2.3) in R, with the significance of differences between survival curves determined by the log-rank test.

### Statistical analyses

Statistical analyses and data presentation in this study were performed using GraphPad Prism 8.0 software by GraphPad Software. Detailed methodologies for the analyses and presentations are specified in the figure legends. Statistical significance was established at P values less than 0.05. In vitro experiments were independently repeated multiple times, yielding consistently similar results as noted in the figure legends. The Mann-Whitney *U* test or the Kruskal-Wallis H test was employed to analyze gene expression variations across normal, tumor, and metastatic tissues or to examine clinical features such as Gleason score, tumor stage, or PSA, depending on the number of groups compared. For microarray-based expression profiling, gene probes with the lowest P values were prioritized. Samples lacking gene expression data or patient survival information were omitted from the analyses. Statistical evaluations were conducted using RStudio (version 1.4.1106) with R version 4.1.0.

## Supplementary information

Supplementary Figures with captions revise-no-mark

Original uncropped images of Western blots performed in current study

Supplementary Table 1-4

Source data for the main figures

Source data available for corresponding supplementary figures

## Data Availability

This study utilized public datasets including GSE21019, GSE6919, GSE3325, GSE6099, GSE21034, and TCGA PRAD. Additionally, for the analysis of ZMYND11 expression levels across various cancers presented in Fig. 1A, datasets GSE7696 (Brain), GSE7803 (Cervix), GSE20842 (Colorectal), GSE23400 (Esophagus), GSE13911 (Gastric), GSE2549 (Mesothelioma), GSE15471 (Pancreas), and GSE21034 (Prostate) were employed. CPGEA RNA-seq data were sourced from http://www.cpgea.com/. All RNA-seq data generated during this study have been deposited in the Gene Expression Omnibus (GEO) under accession codes GSE275451. Raw data and the MaxQuant output for the proteomics have been deposited to the integrated proteome resources (iProX) database under the accession number IPX0009564000. Any other data supporting the findings of this study are available from the corresponding authors upon reasonable request. Relevant URLs include the TCGA data matrix (https://tcga-data.nci.nih.gov/tcga/dataAccessMatrix.htm) and the TCGA Research Network (http://cancergenome.nih.gov/).
