# Supplementary material for: Epigenetic reader ZMYND11 noncanonical function restricts HNRNPA1-mediated stress granule formation and oncogenic activity
Source: Signal Transduct Target Ther. 2024 Sep 28;9:258. doi: 10.1038/s41392-024-01961-7 (PMC11438962; doi:10.1038/s41392-024-01961-7)

Original uncropped Western blots in main and supplementary figures.

Fig.2a

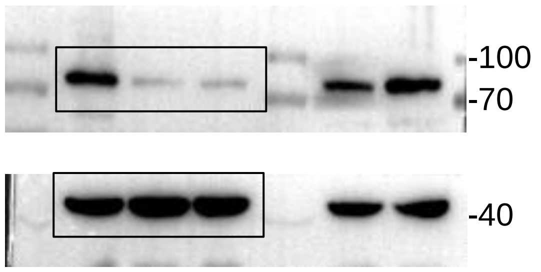

Fig.2i

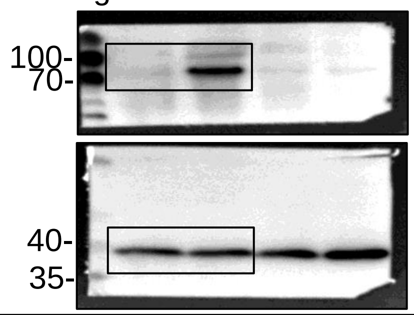

Fig.2p

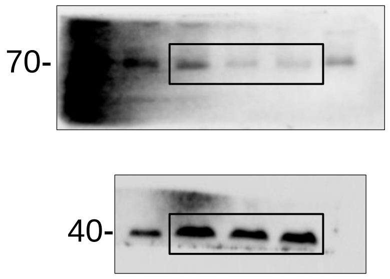

Fig.3c

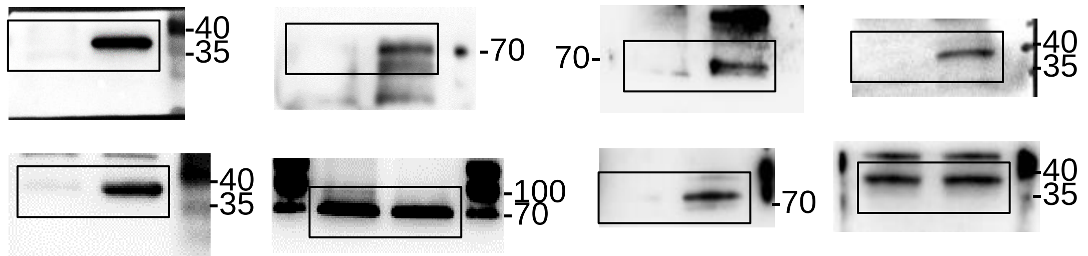

Fig.3d

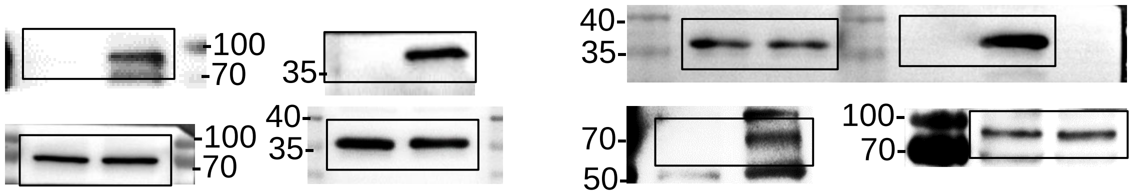

Fig.3g

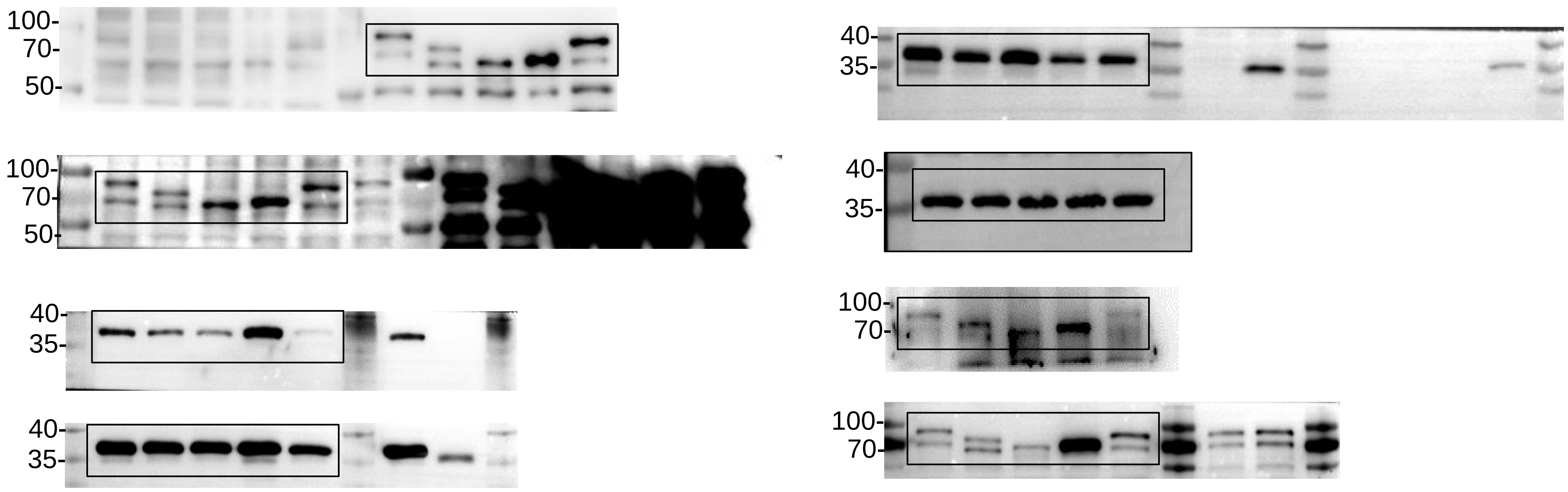

Fig.3j

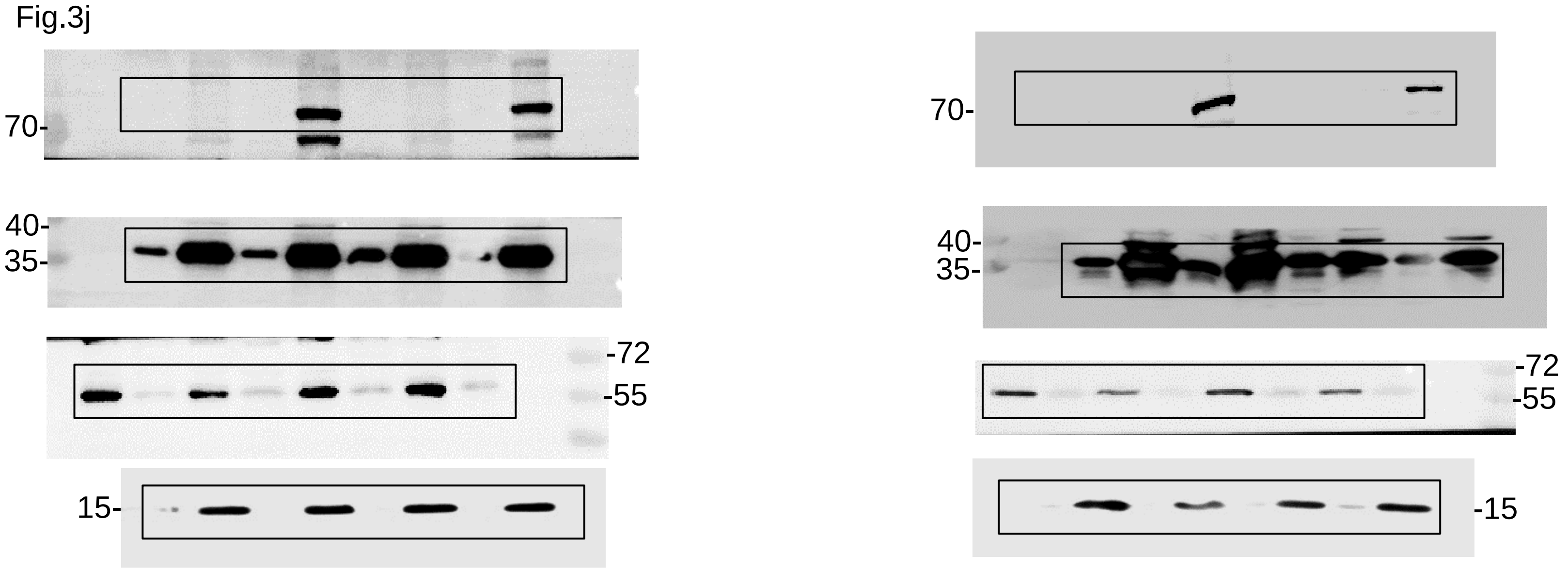

Fig.4a

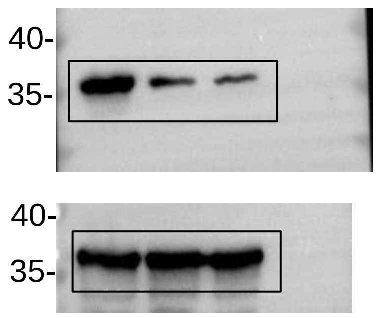

Fig.5a

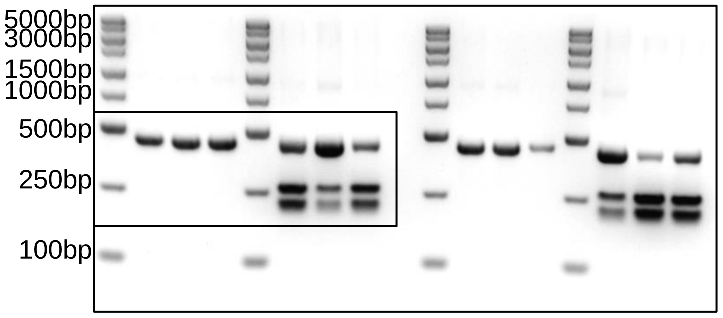

Fig.5b

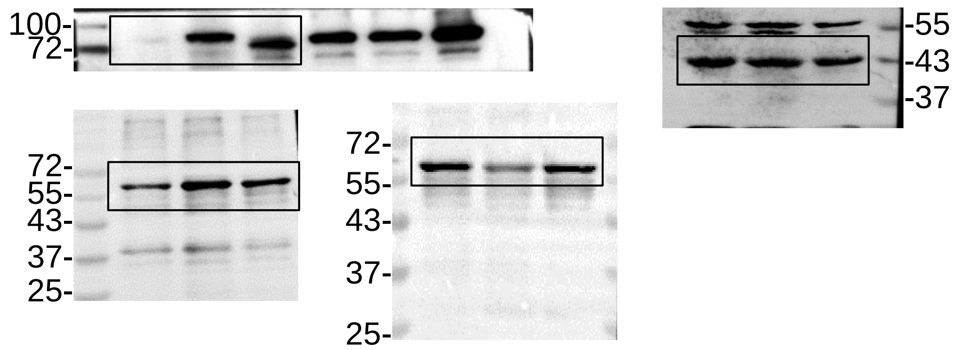

Fig.5c

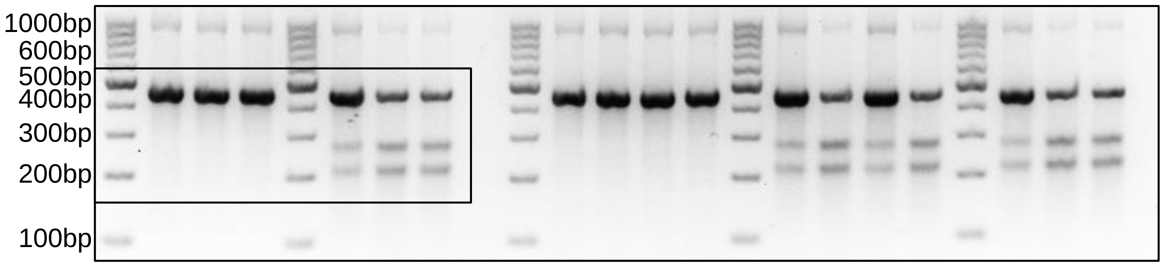

Fig.5d

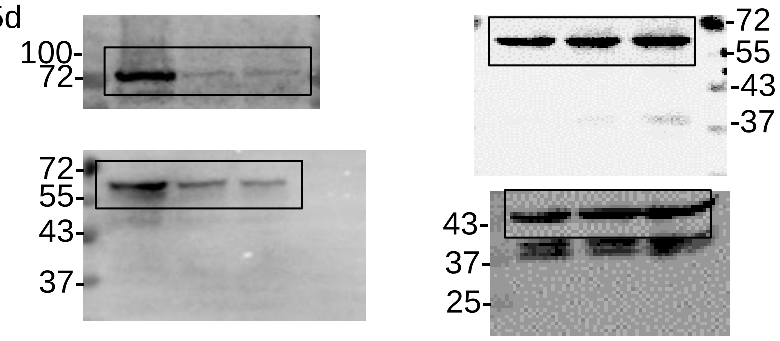

Fig.5e

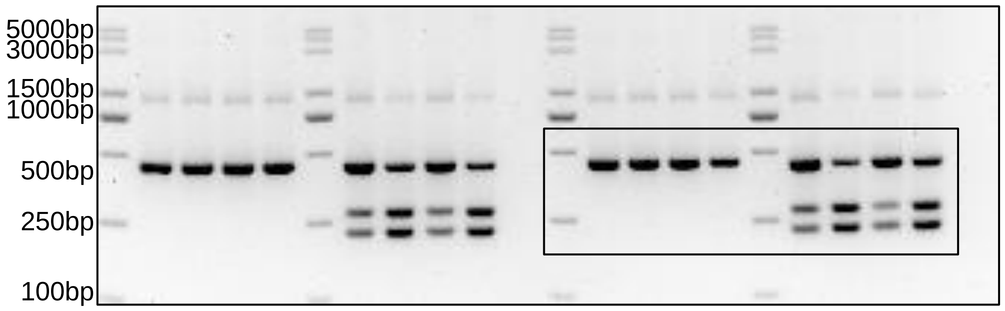

Fig.5f

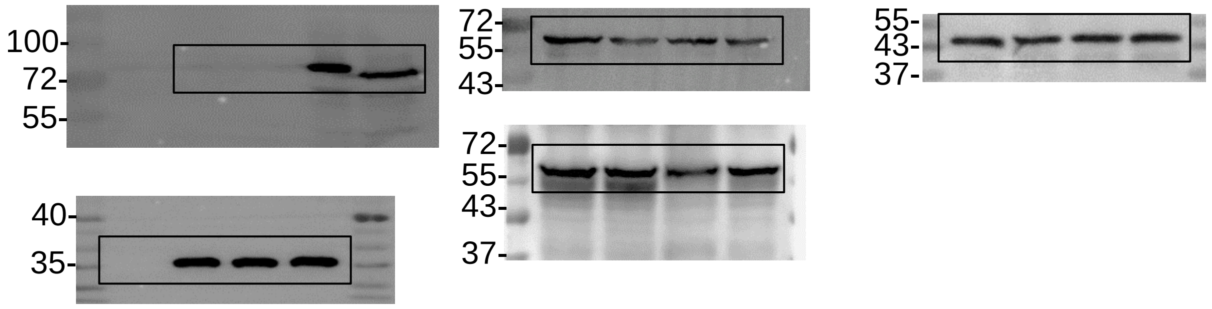

Fig.6b

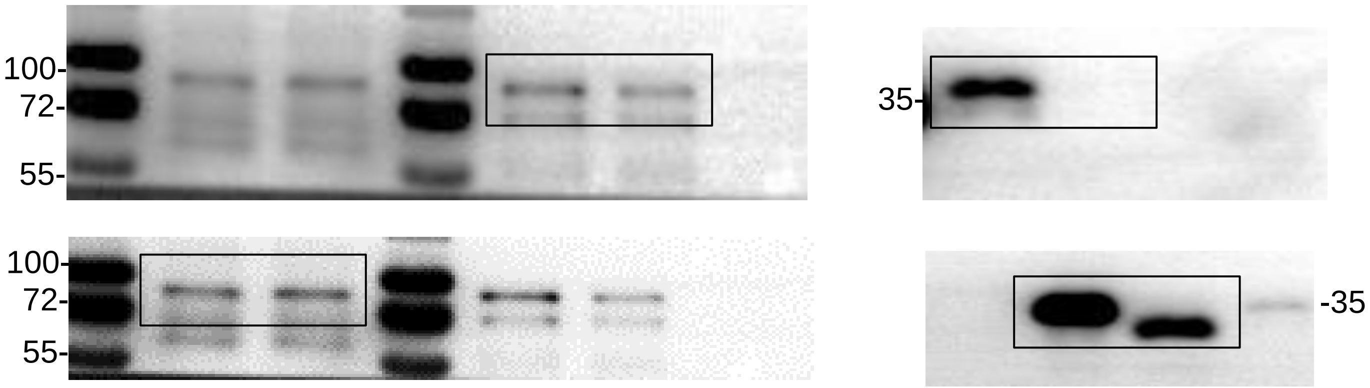

Fig.6h

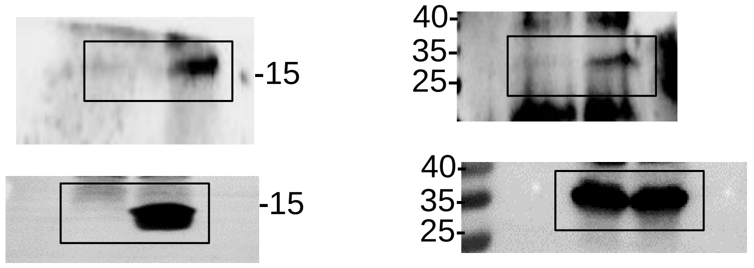

Fig.6c

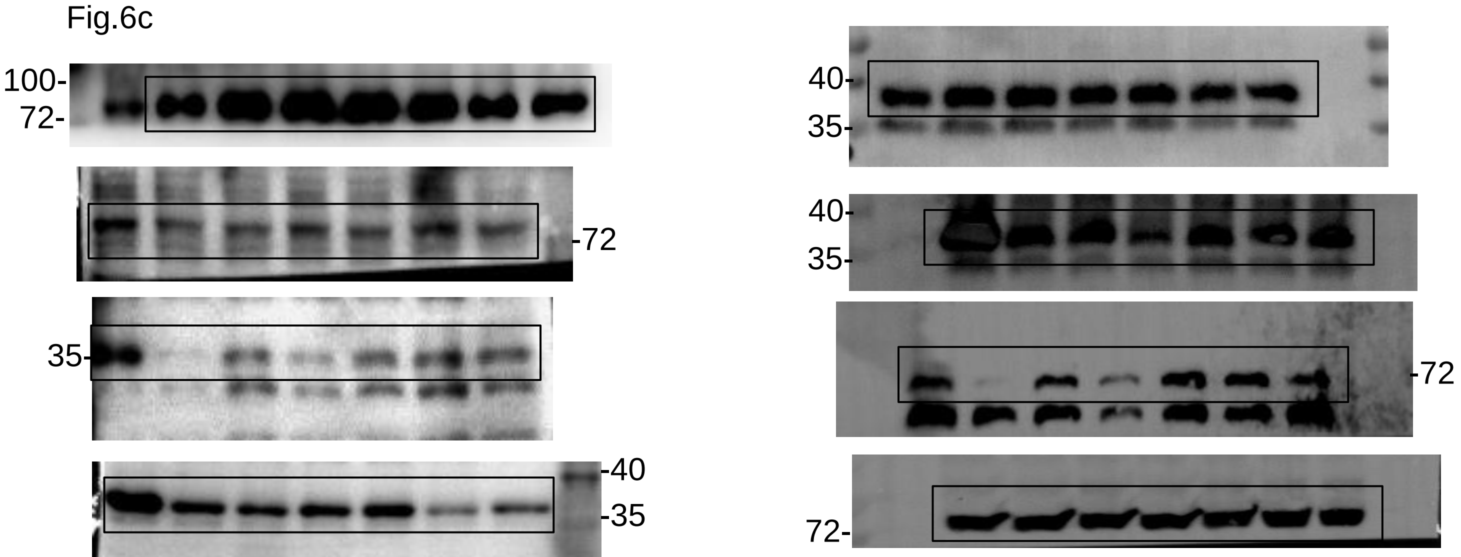

Fig.6i

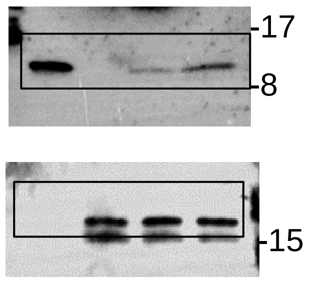

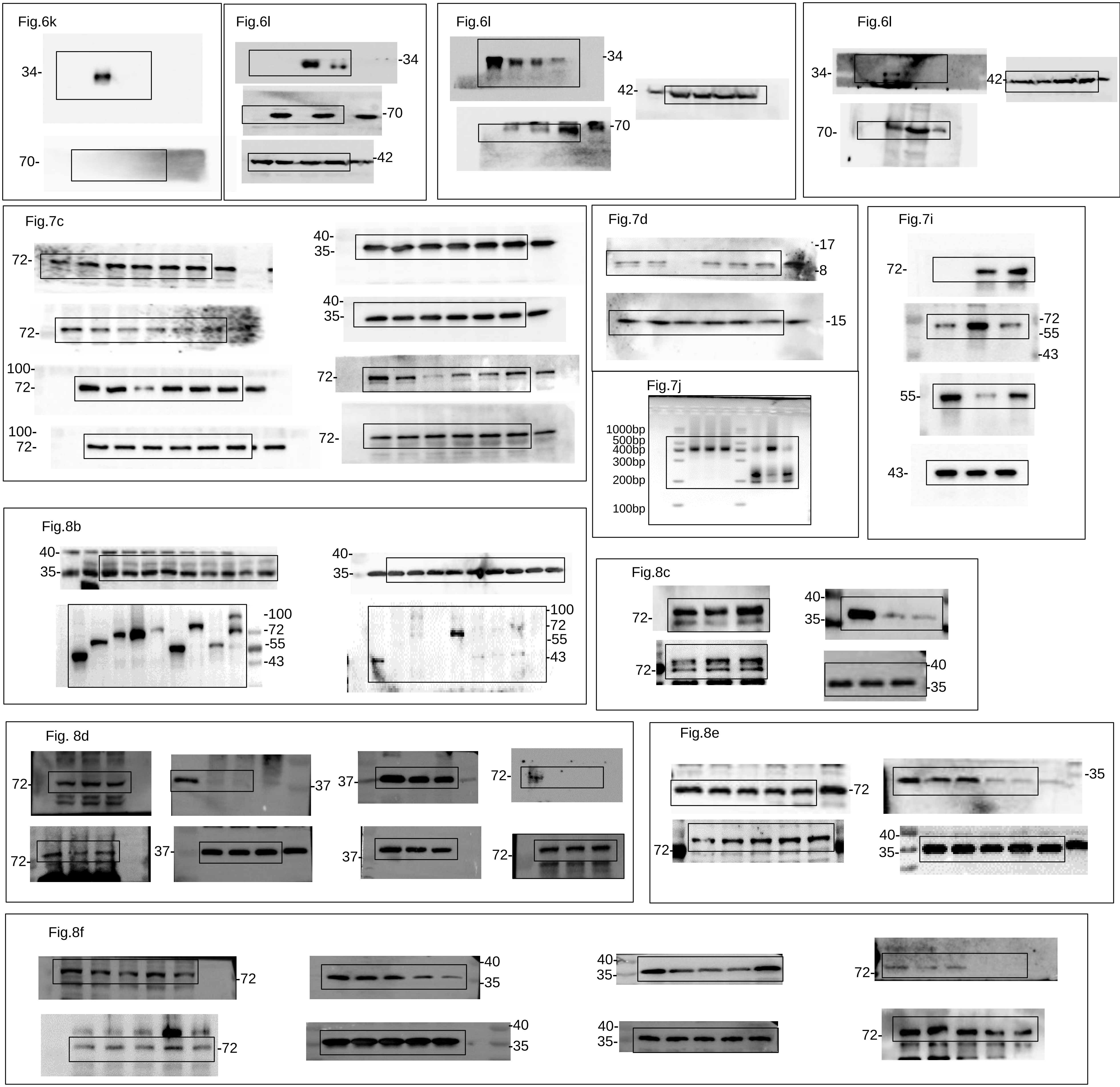

Fig.6k
34-
70-
Fig.6l
-34
-70
-42
Fig.6l
-34
42-
-70
Fig.6l
34-
42-
70-
Fig.7c
72-
72-
100-
72-
100-
72-
40-
35-
40-
35-
72-
72-
Fig.7d
-17
-8
-15
Fig.7j
1000bp
500bp
400bp
300bp
200bp
100bp
Fig.7i
72-
-72
-55
-43
55-
43-
Fig.8b
40-
35-
-100
-72
-55
-43
40-
35-
-100
-72
-55
-43
Fig.8c
72-
72-
40-
35-
-40
-35
Fig. 8d
72-
72-
-37
37-
37-
37-
72-
72-
Fig.8e
-72
72-
-35
40-
35-
Fig.8f
-72
-72
-40
-35
-40
-35
40-
35-
40-
35-
72-
72-

Supp. Fig. 1d

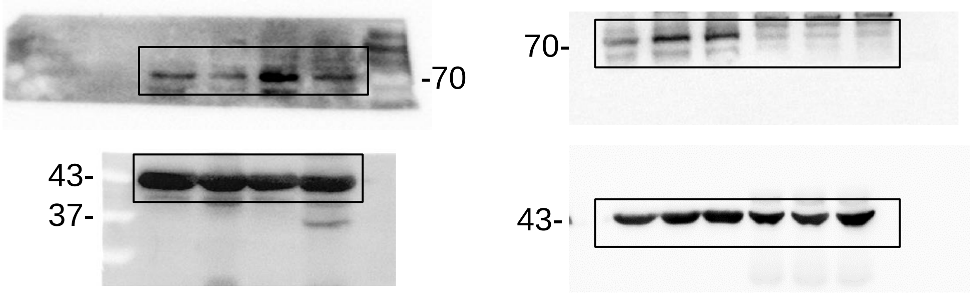

Supp. Fig. 2a

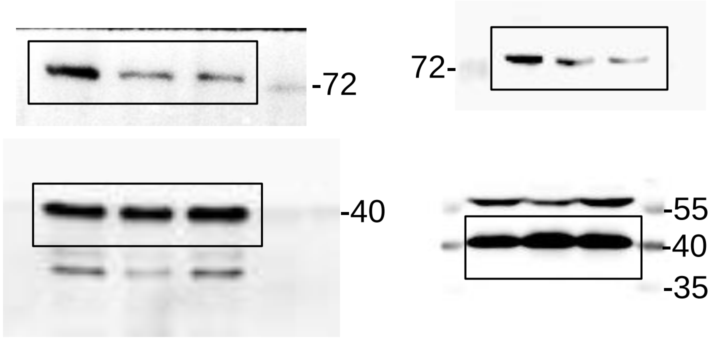

Supp. Fig. 2h

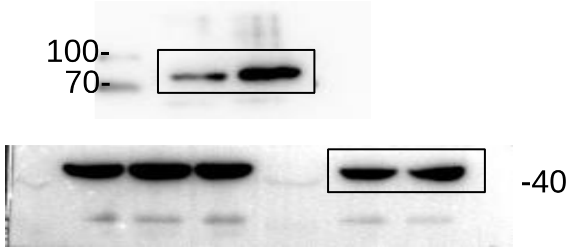

Supp. Fig. 3b

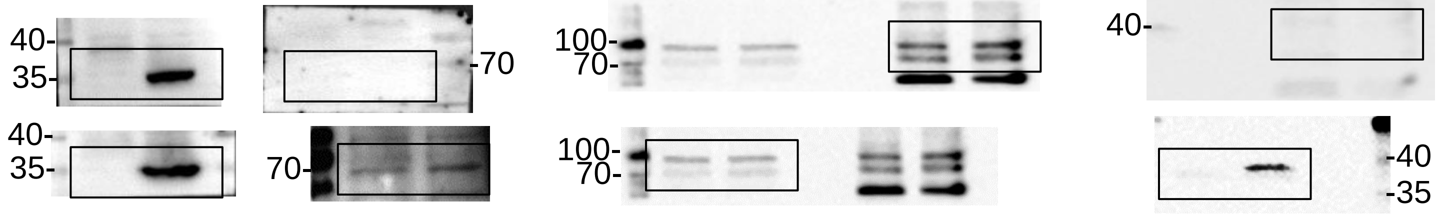

Supp. Fig. 3c

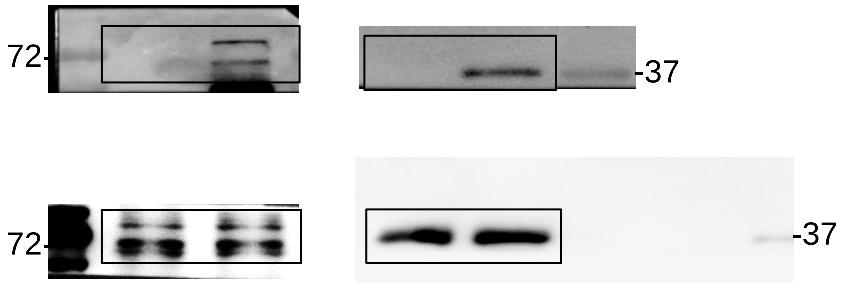

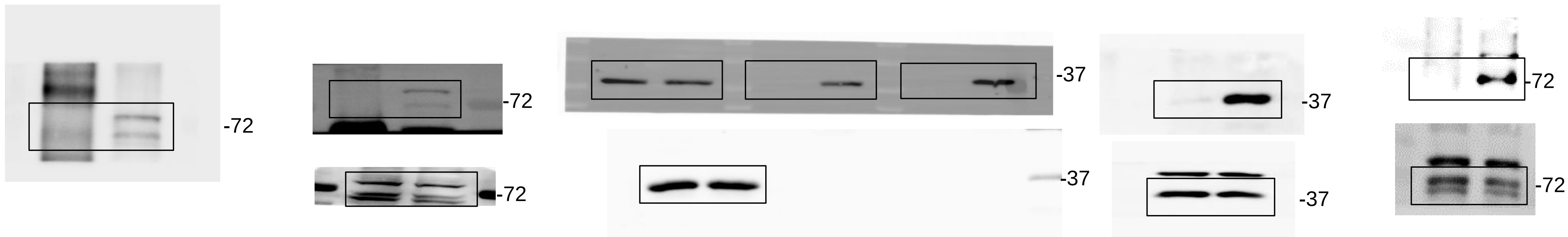

Supp. Fig. 3d

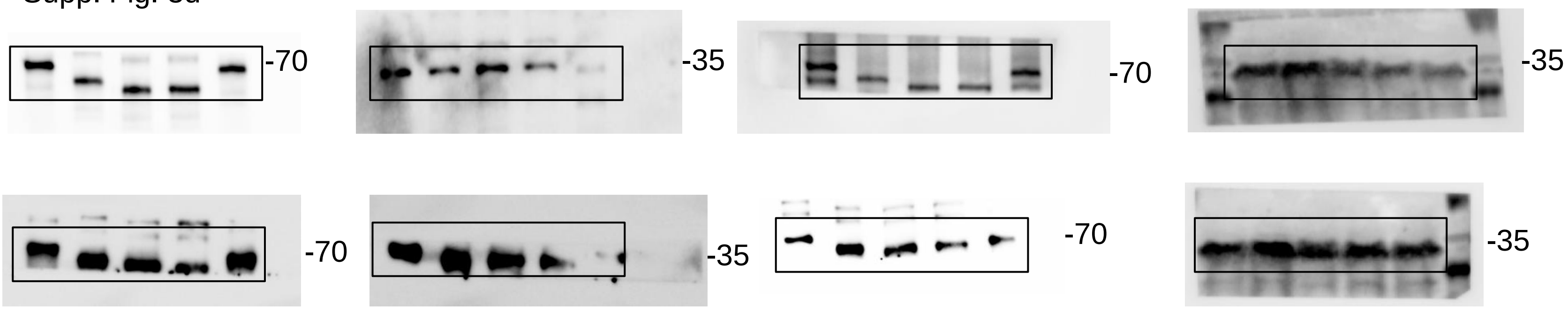

Supp. Fig. 4a

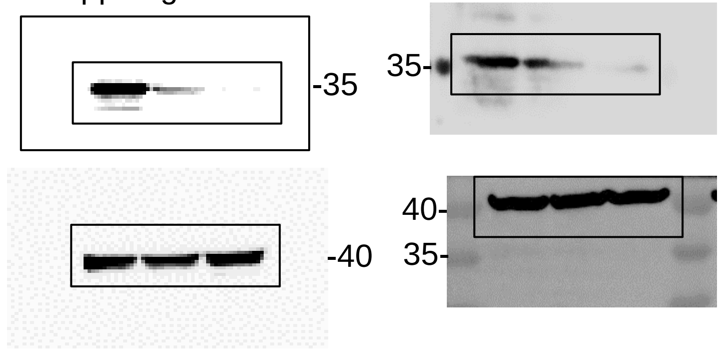

Supp. Fig. 4h

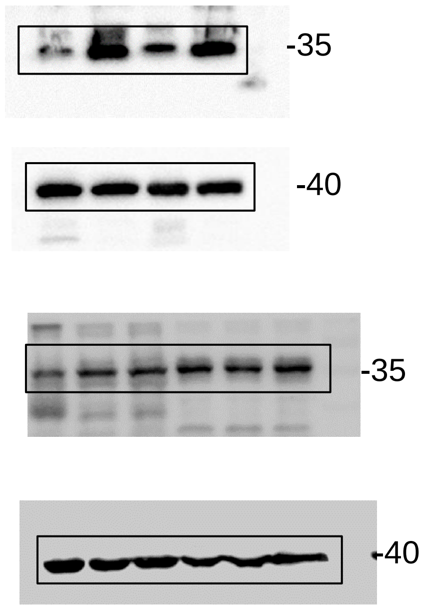

Supp. Fig. 6b

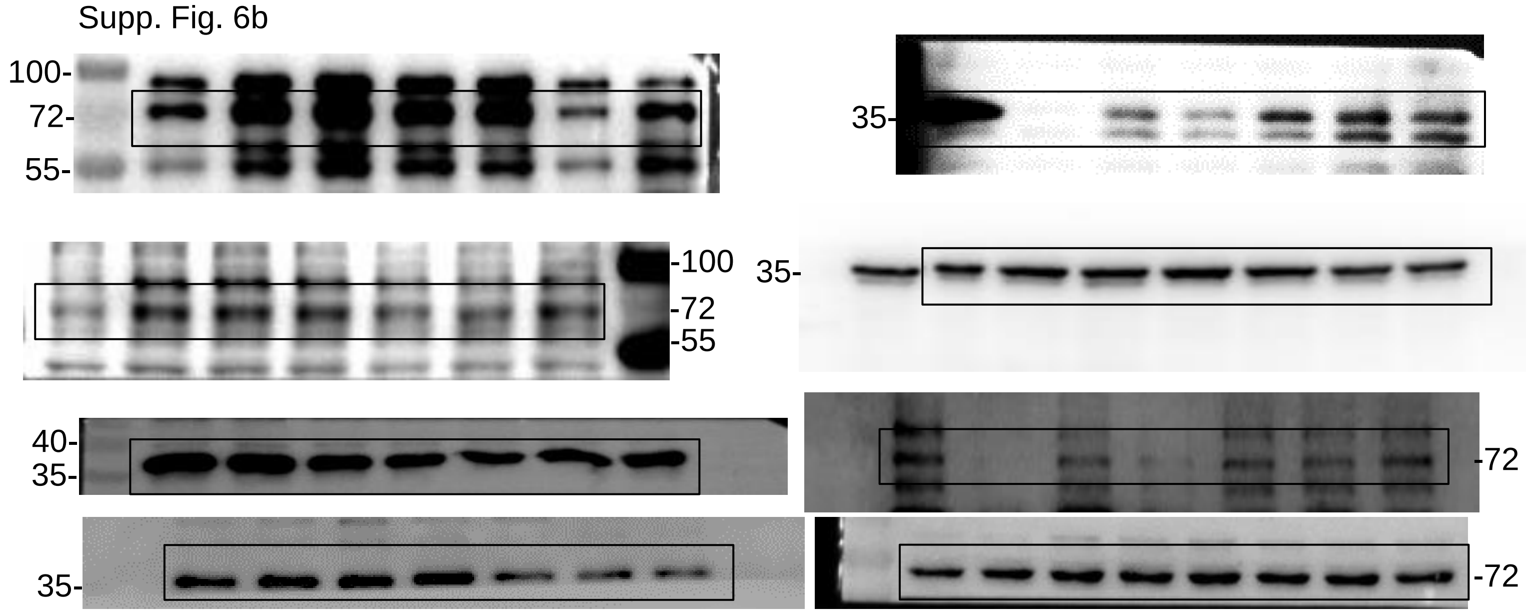

Supp. Fig. 6c

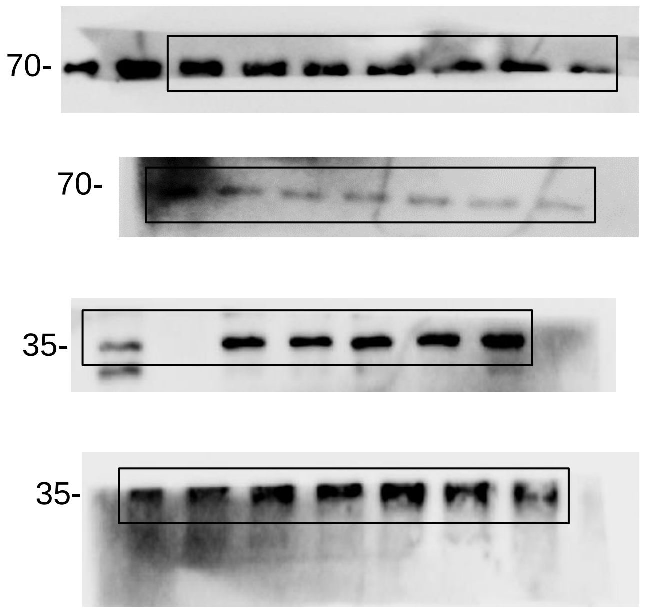

Supp. Fig. 6d

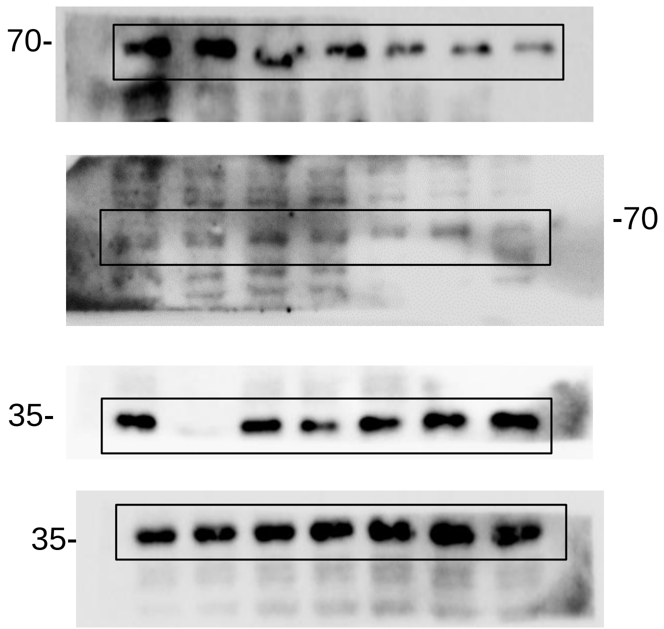

Supp. Fig. 6e

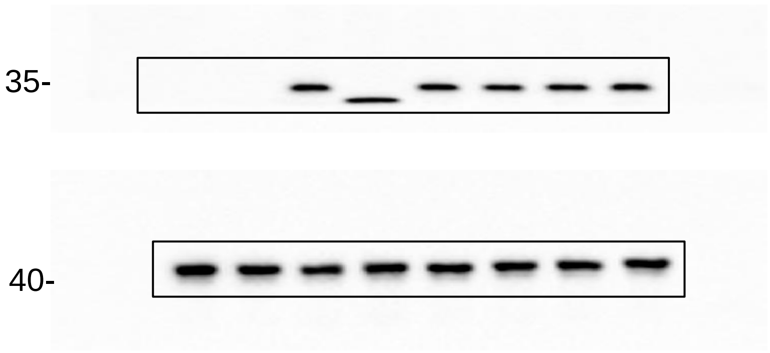

Supp. Fig. 6f

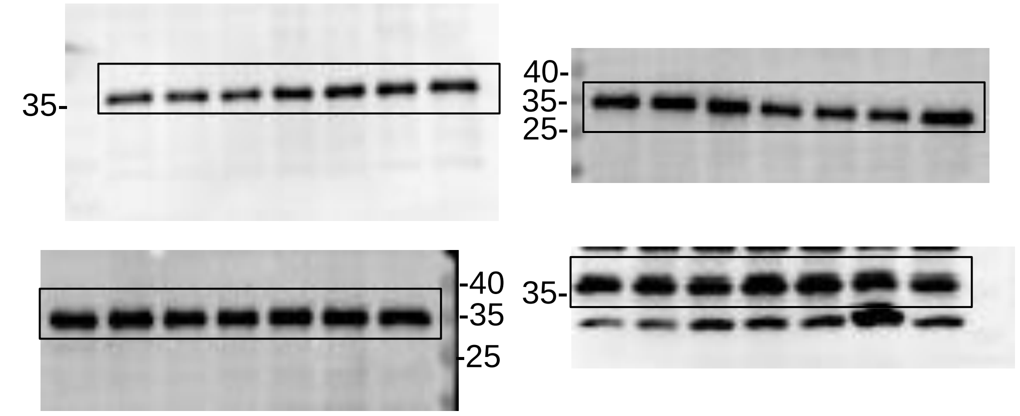

Supp. Fig. 6g

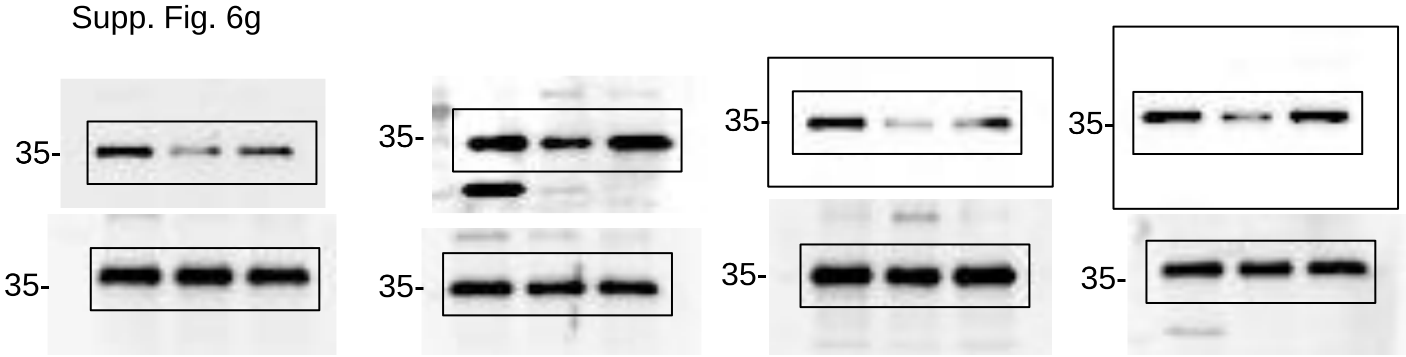

Supp. Fig. 7a

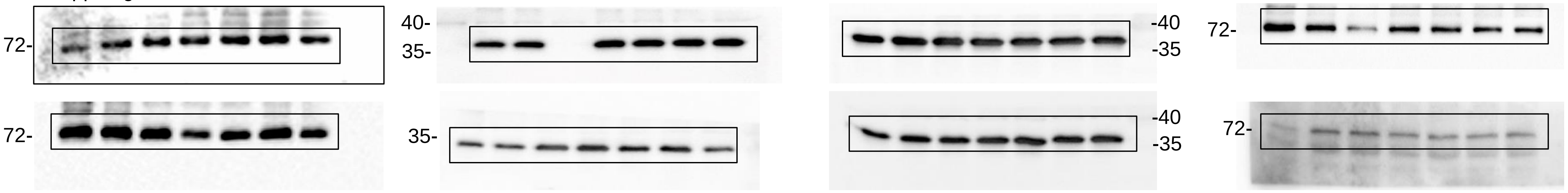

Supp. Fig. 7b

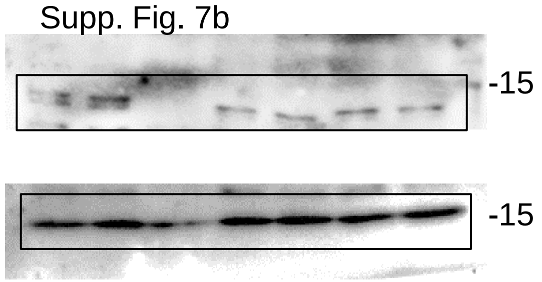

Supp. Fig. 7c

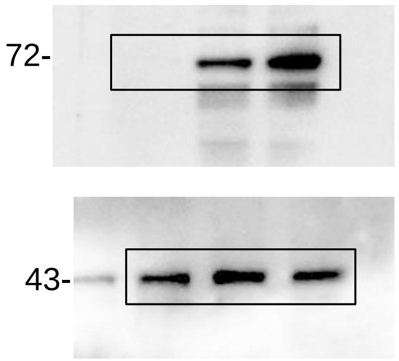

Supp. Fig. 8a

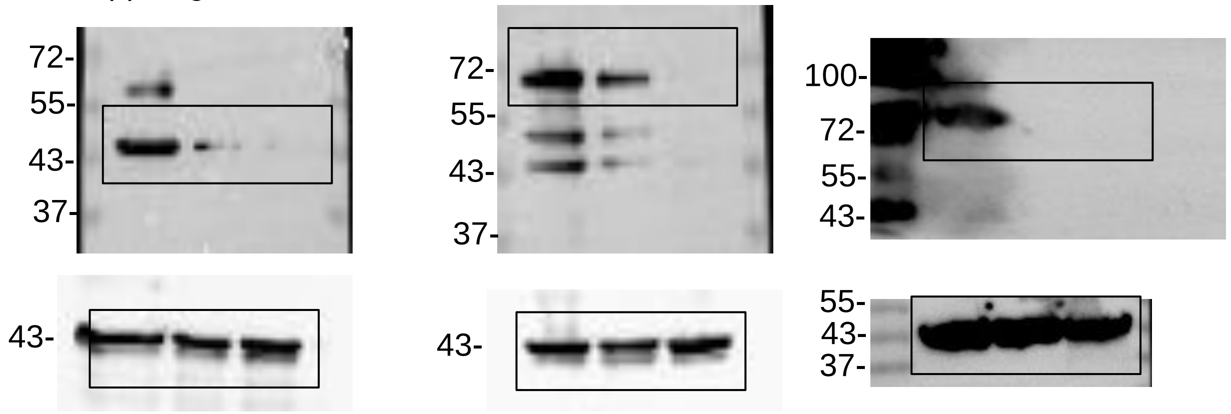

Supp. Fig. 8b

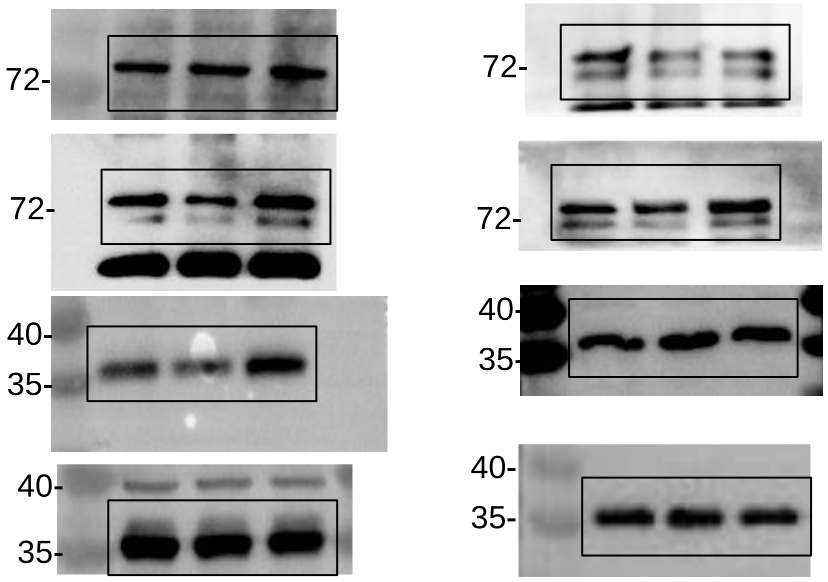

Supp. Fig. 8c

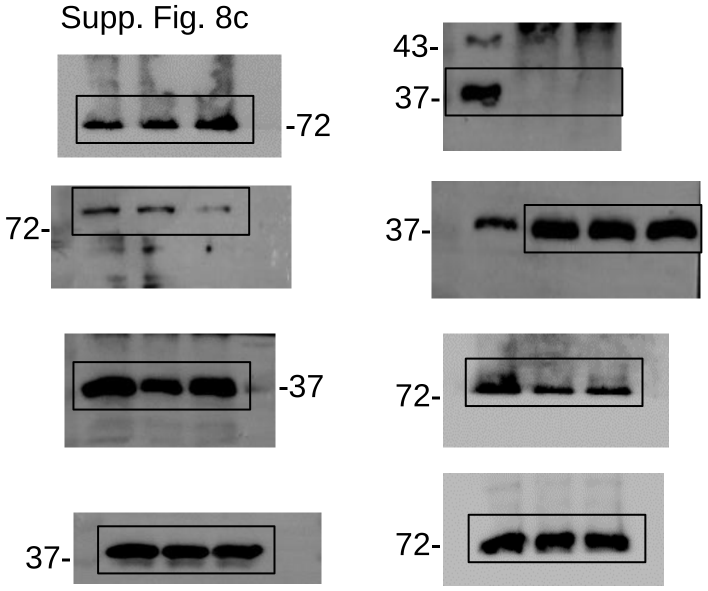

Supplement: Supplementary file 2 — Original uncropped images of Western blots performed in current study [file 41392_2024_1961_MOESM2_ESM.pdf]
